# Multistep loss of catalytic and ligand binding abilities of hexameric purine nucleoside phosphorylase

**DOI:** 10.1038/s41598-026-41204-z

**Published:** 2026-03-02

**Authors:** Marta Narczyk, Agnieszka Bzowska

**Affiliations:** Division of Biophysics, Institute of Experimental Physics, Faculty of Physics, University of Warsaw, Pasteura 5, 02-093 Warsaw, Poland

**Keywords:** Purine nucleoside phosphorylase, Allostery, Catalytic activity, Dissociation constants, Binding fraction, Incompetent fraction, Biochemistry, Biophysics, Chemical biology, Chemistry, Structural biology

## Abstract

It is commonly believed that enzymatic catalysis is such a complex process, that even a small change in any physicochemical property of the enzyme results in a complete loss of the catalytic and ligand-binding capacity of the molecule. Therefore, when the enzyme sample is not fully active, but is electrophoretically pure, the inactive fraction is often considered equal to the non-binding fraction, and constants characterizing ligand binding by such an enzyme and ligand-induced inhibition are determined under this assumption. Here, we present an enzyme, hexameric purine nucleoside phosphorylase, whose gradual loss of a catalytic activity towards natural substrates does not correlate with the loss of the ability to bind ligands, substrates, and inhibitors. The values of dissociation constants characterizing ligand binding depend on the specific activity of the enzyme used in the experiment. Furthermore, there is a stable state of the enzyme, in which it is no longer able to catalyse reaction with natural substrates, but can still catalyse the same reaction if a substrate resembling the transition state is used. The active site conformations of individual subunits in the X-ray structure of this hexameric molecule reflect the presence of intermediate states observed in the enzyme activity decline profiles.

**Supplementary Information:**

The online version contains supplementary material available at 10.1038/s41598-026-41204-z.

## Introduction

Purine nucleoside phosphorylase (PNP, E.C. 2.4.2.1) is the enzyme involved in the metabolism of purine bases, nucleosides and nucleotides. It is one of the key proteins in the so-called purine salvage pathway, that enables cells to recover purine bases and pentose-1-phosphate from used nucleosides. PNP catalyses the reversible phosphorolytic cleavage of the glycosidic bond of ribo- and 2’-deoxyribo purine nucleosides as follows:

1 $$\beta-{\mathrm{purine}}\;{\mathrm{nucleoside}} + {\mathrm{orthophosphate}} \leftrightarrow {\mathrm{purine}}\;{\mathrm{base}} + \alpha\mathrm{-}{\mathrm{D}}\mathrm{-}{\mathrm{pentose}}\mathrm{-}{\mathrm{1}}\mathrm{-}{\mathrm{phosphate}}$$

PNPs from various organisms are divided into two subfamilies with very low sequence similarities: homotrimers (in terms of the amino acid sequence of subunits) mainly of mammalian origin, and homohexamers found mainly in prokaryotic organisms. Differences are observed not only in the sequence and the oligomeric architecture, but also in the specificity of enzymes from these two subfamilies. Trimeric PNPs accept only 6-oxopurines and their nucleosides as substrates, hence their natural substrates are inosine and guanosine (and their 2’-deoxy counterparts), while hexameric enzymes have much broader specificity and in particular their substrates include 6-aminopurines and their nucleosides^1^.

PNPs from various organisms are widely studied for their possible medical applications, because they are considered as potential targets for drugs in immunosuppressive, antitumour, and antiparasitic therapies. Discovery of Giblett et al.^2^ that PNP deficiency in humans hampers cellular immunity, while leaving normal humoral response, suggested that inhibitors of human PNP may act as selective immunosuppressive agents. After many years of intense studies, one such inhibitor, immucillin H (forodesine), the inosine analogue (with the C-C instead of the N-C glycosidic bond and iminosugar instead of the ribose) was accepted in Japan for the treatment of relapsed/refractory peripheral T-cell lymphoma^3^.

Differences in substrate specificity of trimeric and hexameric enzymes of the PNP family led to the idea of gene therapy for solid tumours, in which hexameric PNP is used as a prodrug-activating enzyme^4,5^. Finally, lack of *de novo* nucleoside synthesis pathway in some bacteria, e.g. in *Helicobacter pylori*^6^, and in some parasites like e.g. in *Plasmodium* causing malaria^7^ makes enzymes of the purine salvage pathway, including PNP, potential targets to design drugs to combat these pathogens, e.g.^8,9^.

In addition to the important medical applications of PNPs and their inhibitors, enzymes from this family are also remarkable molecular machines and as such pose a challenge for molecular biophysics research. In particular, they are excellent models to broaden our understanding of a nontrivial enzymatic mechanisms since all PNPs, both hexameric and trimeric, show complex kinetic characteristics, which was noted immediately when these enzymes were identified and characterized, e.g.^10,11^. Namely, not the Michaelis-Menten, but the four-parameter kinetic equation is necessary to describe kinetics of these enzymes vs. phosphate, and also vs. nucleosides when phosphate concentration is not saturating. This suggests that PNPs has two binding sites with different affinities for substrates, or that there is a negative cooperativity between enzyme subunits (see^1^). However, it was shown that for trimeric PNPs this complex kinetic behaviour is caused by the unusually slow, hence strongly rate-limiting, dissociation of the products (guanine and hypoxanthine), and by a dual function of the phosphate (with phosphate acting as a substrate and as a modifier helping in the release of a purine base after glycosidic bond cleavage)^1^.

**Fig. 1:**
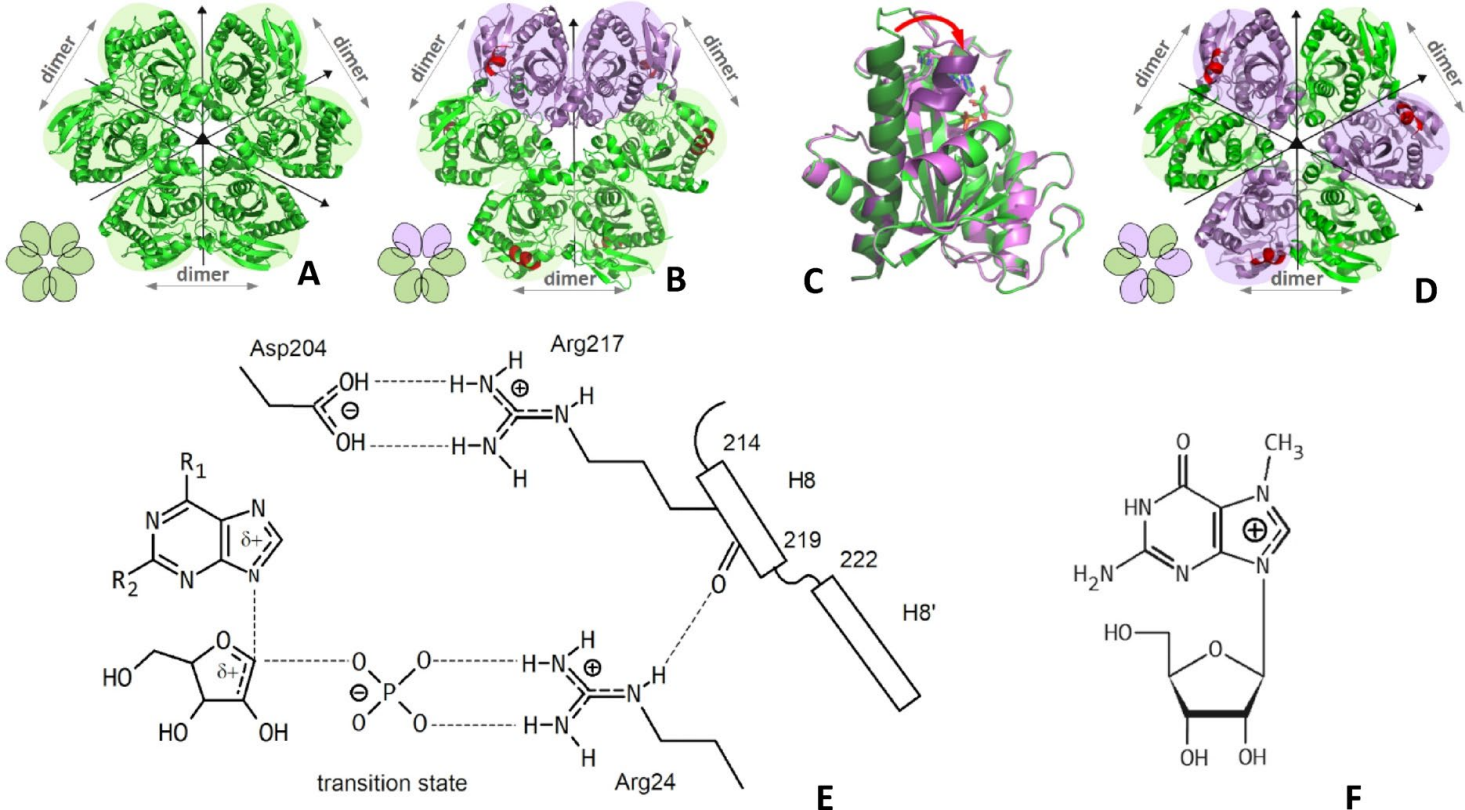
Structure of the *E. coli* PNP (panels **A**–**D**), its mechanism of catalysis (panel **E**), and the chemical structure of 7-methylguanosine (panel **F**), the unusual substrate of PNP, resembling the transition state of the enzyme due to positive charge on the imidazole ring of the purine base. Panel (**A**): the apo form of the enzyme with all six subunits of the hexameric molecule per asymmetric unit, space group P2_1_. Structure of all subunits is the same with the active site in the open conformation^12^ (PDB 1ecp). The molecule has 32 point group symmetry. Panel (**B**): fully liganded enzyme form, with phosphate and the non-cleavable analogue of the nucleoside substrate (formycin B, with the C-C glycosidic bond), in a hexagonal space group P6_1_22, with a half of the hexamer in the asymmetric unit. Active site of four subunits is in the open (green) and of the other two subunits in the closed conformation (violet), which leads to the presence of two open-closed dimers and one open-open dimer^13^ (PDB 1a69). Panel (**C**): overlaid two enzyme subunits, one in the open and one in the closed conformation of the active site^13^ (PDB 1a69) showing the differences between these two active site conformations: the segmentation of the helix H8, the movement of its N-terminal part (shown in darker shades of green and violet, respectively), and the loop, towards the active site pocket (indicated by the red arrow). Panel (**D**): the fully liganded enzyme form (with phosphate and 6-methylformycin A) in a tetragonal space group P4_1_2_1_2, in which a full hexamer in present the asymmetric unit. This structure reveals three open-closed dimers, and it was obtained using the enzyme sample with the highest activity ever observed^14^ (PDB k9s). Panel (**E**): transition state of the *E. coli* PNP, with the protonated imidazole ring of the purine base and ribooxocarbenium ion character of the pentose. Binding of phosphate stabilizes the H8 helix in two segments, hence the closed conformation of the enzyme active site, due to hydrogen bonds between phosphate-Arg24 and Arg24-Arg217. The N-terminal segment of the helix H8, residues 214–219, closes the entrance to the active site pocket and brings the guanidinium group of Arg217 in contact with the acidic Asp204, resulting in proton transfer to the purine base position N7 and the formation of the salt bridgeArg217-Asp204. The positive charge thus created on the purine base leads to the ribooxocarbenium ion formation at the pentose^14^. Panel (**F**): the unusual PNP substrate, 7-methylguanosine, which, due to methylation of the position N7 of the purine base, already bears a positive charge on the imidazole ring of the purine base, hence protonation by the enzyme is not necessary for the catalysis of the glycosidic bond cleavage.

Whereas for PNP from *E. coli*, which is the main subject of this study, molecular mechanism causing its complex kinetics behaviour turned out to be completely different. This enzyme is a homohexamer, in terms of amino acid sequence of monomers, and in the apo form the three-dimensional structure of all subunits is the same. However, as shown on the Fig. 1 (panel A), the whole molecule may be regarded as a trimer of dimers. The active sites of subunits comprising a dimer are located face to face, and mutually donate to each other side chains of His4 and Arg24, which are necessary to complete the active site of the neighbour^12^. This suggests that the basic catalytic unit of the protein is a dimer and points to a negative cooperativity within a dimer as a reason for the complex kinetic behaviour of this enzyme. It was therefore rather puzzling when the first crystal structure of the liganded *E. coli* PNP was published^13^ and yielded a molecule with two, as expected, unsymmetrical dimers, each with one open and one closed active site conformation, but with the third dimer being a fully symmetric having both active sites in the open conformation (see Fig. 1, panel B). Closing of the active site is a result of the segmentation of helix H8 and movement of its N-terminal part towards the active site pocket (see Fig. 1, panel C). The most obvious explanation of such a difference among the dimers seemed to be that in this case the crystal symmetry forces one of the three dimers to be symmetric (with disordered C-terminal part of the protein). Indeed, the next liganded structure of this enzyme^14^ revealed three unsymmetrical open-closed dimers (Fig. 1, panel D). Over the years, however, it turned out that this was the only structure of this enzyme with such symmetry, while the structure with two unsymmetrical and one symmetrical dimer is typical for *E. coli* PNP, recombinant and not recombinant, complexed with various ligands (e.g.^13,15,16,17^). Such an enzyme architecture is observed also in the crystallographic X-ray structures in which the all six subunits forming the molecule are symmetrically independent, hence it would be permissible to observe possible differences between the subunits forming the open-open dimer, if any existed^18^. Moreover, it is also observed in the structure of hexameric PNP from other sources^19^.

Closing of the enzyme active site pocket, which is a necessary step in the catalysis conducted by the *E. coli* PNP, is triggered by binding of phosphate, which is one of the enzyme substrates. Segmentation of the helix H8 and its movement towards the entrance of the active site brings the catalytic Asp204 close to the Arg217 (see Fig. 1, panel E), which in turn enables the proton transfer from Asp204 to the position N7 of the nucleoside substrate. This leads to the charge relocation between purine base and pentose producing the ribooxocarbenium ion of the latter. Hence, positively charged imidazole ring of purine and positively charged pentose represent the PNP transition state of the phosphorolytic cleavage reaction of the purine nucleosides glycosidic bond^14,18^. It is worth to mention here, that PNP accepts as a substrate 7-methylguanosine (Fig. 1, panel F), and 7-methylinosine, which, due to N7 methylation, bear a positive charge on the imidazole ring at neutral pH^20^. This suggests that the open-open dimer might be not active, at least vs. natural substrates bearing no positive charge at neutral pH. The above hypothesis regarding the catalytic inactivity of the open-open dimer is supported by binding studies of the Y160W *E. coli* PNP mutant. Experimental results from multiple techniques consistently show that dimers indeed could be not equivalent, as three binding constants are necessary to describe the binding process of phosphate and of the nucleoside, despite the fact that a main catalytic unit of the hexameric PNP seems to be a dimer^21^. In this paper we try to solve this puzzle and look more deeply into the molecular mechanism of communication between all six subunits forming a biologically active *E. coli* PNP, which controls closing and opening of the enzyme active sites. We combine some perplexing results obtained over last decades with the new data currently collected to resolve these unexplained results and build a coherent picture.

## Results and discussion

### Elusive specific activity of *E. coli* PNP

**Fig. 2:**
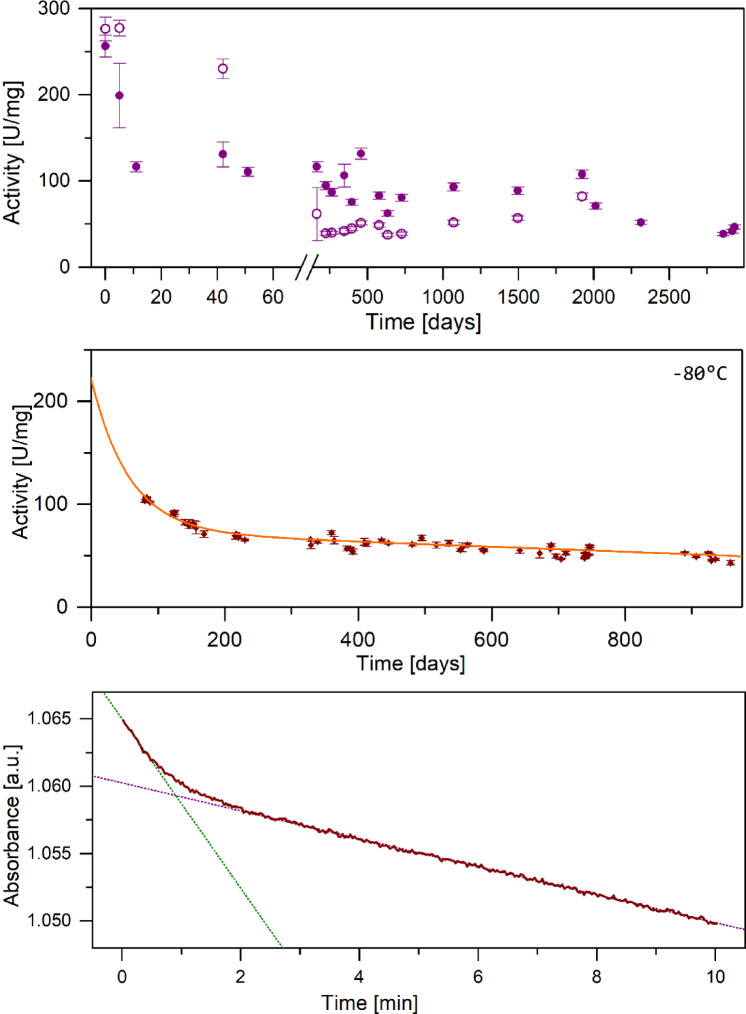
Multiphase activity profile of the *E. coli* PNP. Upper panel: Change in the specific activity vs. Ino occurring during storage of the partially purified sample of the *E. coli* PNP obtained as a gift form Dr. George Koszalka; open circles: samples, kept frozen at -80 °C and thawed for purification; closed circles: sample just after purification on the affinity chromatography column (with the Sepharose activated with formycin B) and concentration. Measurements of the enzyme specific activity were carried out at standard conditions (see “Materials and methods” Section). The SDS-PAGE of the sample after purification with the specific activity vs. Ino 103.2 ± 4.0 U/mg, and of the sample with the specific activity vs. Ino 75.0 ± 0.5 U/mg, are shown on Fig. S1 in the Supplementary information. The later sample of the enzyme, with the lower specific activity, is also electrophoretically pure. Middle panel: Decrease in the specific activity vs. Ino occurring during storage of the purified recombinant *E. coli* PNP at -80 °C in 10 mM Tris buffer pH 7.4 (points) with errors calculated as a mean standard deviation. Measurements of the enzyme specific activity were carried out on freshly thawed samples, at standard conditions (see “Materials and methods” Section). The line shows the result of fitting of Eq. (4) to the activity decline data. Lower panel: Reaction curve of m^7^Guo phosphorolysis at standard conditions (see “Materials and methods” Section) catalysed by the 1.2 nM of the recombinant *E. coli* PNP double mutant Arg24Ala/Arg217Ala showing initial, quick phase with a specific activity of about 221 U/mg, followed by a slower phase with the typically observed specific activity of about 37 U/mg. Inserts show residuals of the strait line fitting to both phases.

Our long-term project devoted to various aspects of *E. coli* PNP catalysis started many years ago^22^, and from the very beginning some of the measurement results were unusual, difficult to understand and interpret, and even gave the impression of artifacts. The project began to develop fully when we have obtained a sample of a purified enzyme with activity of 117 U/mg, and later a huge amount of the partially purified one with a declared activity of about 56 U/mg, both due to the courtesy of Prof. Thomas Krenitsky and Dr. George Koszalka from the Wellcome Research Laboratories (Research Triangle Park, NC, USA). We used to purify the enzyme from the latter sample to homogeneity by the affinity chromatography utilizing Sepharose activated with the PNP inhibitor, formycin B^23^. The partially purified enzyme preparation stored at -80 °C, over approximately 5 years when we were working with this sample, in principle had a specific activity vs. inosine consistent with the declared one, namely 49.6 ± 3.5 U/mg. However, during the first 2 months of our work with this sample we have noticed a strange phenomenon. Activity of the enzyme, freshly diluted for the activity test done before each purification, was as high as 224.2 ± 14.0 U/mg (average from all experiments done in this period), but after dilution was decaying within several minutes to the expected activity of about 50 U/mg. Enzyme samples, freshly purified on the formycin B activated Sepharose, were pure on the SDS PAGE and had specific activity 172.2 ± 9.9 U/mg, hence lower than that of the partially purified freshly diluted preparation (Fig. 2, upper panel). This effect occurred over about 2 months only, but consistently in every purification run performed in this period, and in the hands of more than one experimenter. Therefore, it is unlikely to be an artifact. After this short period of about 2 months, over next about 5 years, the enzyme before purification had a mentioned activity of 49.6 ± 3.5 U/mg, while just after purification 92.2 ± 2.2 U/mg. These activities are in line with the SDS PAGE results of the samples prior and after purification, and are also in line with the information from Dr. George Koszalka that the enzyme preparation purity is of about 60%.

Furthermore, we noticed something resembling this unexpected behaviour, i.e. more than one phase in the activity over time profile, also when we started to work with the recombinant *E. coli* PNP constructed by our group^18^. Freshly purified protein has specific activity vs. inosine slightly above 100 U/mg. Immediately after purification, we freeze small aliquots of PNP at -80 °C, each of which is entirely used for individual experiments to avoid multiple thawing and refreezing cycles. Before performing each experiment, immediately after thawing the sample, we always determine its specific activity. We have noticed that the activity declines steadily over time of enzyme storage at -80 °C, until it reaches the level of about 70 U/mg. The SDS-PAGE of such a sample and that of the freshly purified enzyme are shown on Fig. S1 in the Supplementary information. It is visible that the enzyme samples with the specific activity vs. inosine 103.2 ± 4.0 U/mg and 75.0 ± 0.5 U/mg, are electrophoretically pure.

Thereafter, the rate of decline slows down, resulting in a clearly biphasic curve of specific activity decay during this time period (Fig. 2, middle panel). This particular sample due to some organizational reasons was stored (in aliquots) at -80 °C for about three months before used for intensive studies. The sample was very big so last aliquots were thawed and used after almost three years. The activity decays curve is described by an exponential decay with half-time 56.7 ± 5.6 days, followed by a linear phase with slope 0.024 ± 0.002/day (which is probably also exponential, but was followed too shortly to notice the curvature). The initial activity from extrapolation of the exponential part to time 0 is about 220 U/mg, while from extrapolation of the slow phase to time 0 is 73.3 ± 1.5 U/mg.

The standard assay for the PNP activity uses inosine as a substrate, and is a coupled reaction, in which hypoxanthine, the product of the of inosine phosphorolysis, is oxidized by the xanthine oxidase to form uric acid^11,24^:

$${\mathrm{inosine}} + {\mathrm{orthophosphate}} \leftrightarrow {\mathrm{hypoxanthine}} + {\alpha}\mathrm{-}{\mathrm{D}}\mathrm{-}{\mathrm{ribose}}\mathrm{-}{\mathrm{1}}\mathrm{-}{\mathrm{phosphate}} \leftrightarrow {\mathrm{uric}}\;{\text{acid }}+ {\alpha}\mathrm{-}{\mathrm{D}}\mathrm{-}{\mathrm{ribose}}\mathrm{-}{\mathrm{1}}\mathrm{-}{\mathrm{phosphate}}$$

This assay is not very convenient for the inhibition studies of PNP, because the inhibition observed may arise also from interactions of the tested compound with xanthine oxidase. Therefore, we developed a direct spectrophotometric assay, that may be used in the absorption as well as in the fluorescent variant, based on the phosphorolysis of 7-methylinosine or 7-methylguanosine as a PNP substrate^20,22^. Both of these nucleosides carry a positive charge on the imidazole ring (Fig. 1, panel F), hence their protonation by the enzyme seems not to be necessary^14^. We routinely use this direct assay (e.g.^25,26^), however always specific activity vs. inosine is also determined as a control of the enzyme condition. To our great astonishment, several times it happened that the *E. coli* PNP lost activity vs. inosine but was still fully active vs. m^7^Guo. This was certainly not an artifact, because we observed phosphorolysis of m^7^Guo when we added this substrate to a cuvette with a reaction mixture prepared to monitor inosine phosphorolysis, but in which the reaction with inosine did not occur. Moreover, we have observed the same effect with the hexameric phosphorylase from S*ulfolobus solfataricus*, which has a three dimensional structure very similar to that of *E. coli* PNP^27^.

Furthermore, phosphorolysis of m^7^Guo catalysed by the double mutant of *E. coli* PNP, Arg24Ala/Arg217Ala, was found to be biphasic (Fig. 2, lower panel) with the specific activity of the fast and the slow phases of about 221 U/mg and 37 U/mg, respectively. However, this phenomenon was observed only with the freshly purified enzyme, in line with the fact that the slower phase exhibits activity typically observed for this mutant, 46.9 ± 5.2 U/mg^18^. The fast phase lasts much longer than the single turnover of the reaction (µM of the substrate is consumed while enzyme concentration is in the nM range), hence product inhibition is not the explanation of this phenomenon.

### Catalytically inactive protein is able to bind ligands

**Fig. 3:**
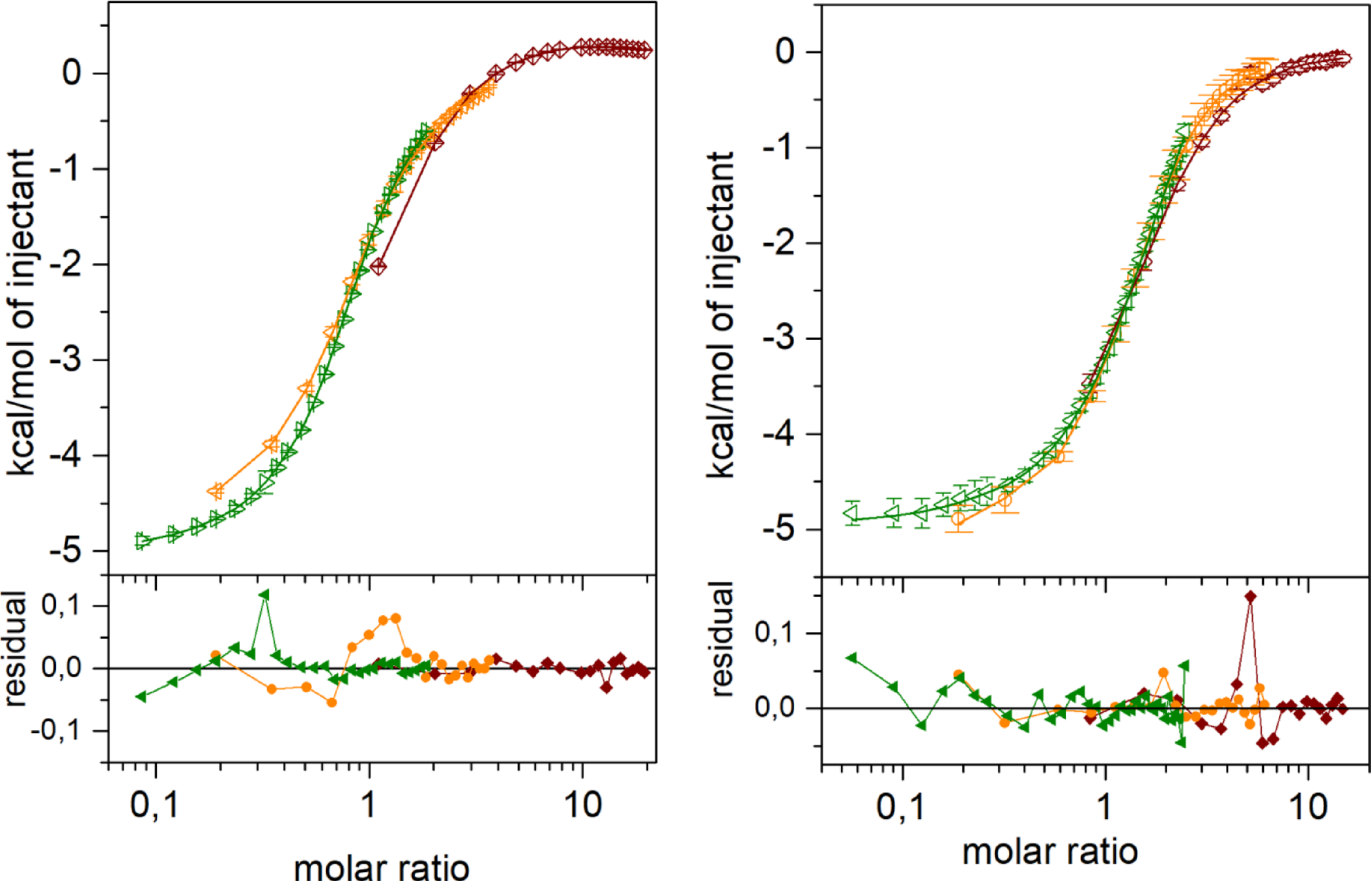
Isothermal titration calorimetry studies of ligand binding by *E.* coli PNP. Titrations of the recombinant *E. coli* PNP with phosphate (left panel), and of the enzyme- phosphate complex with formycin A (right panel). Data with errors (points with error bars), global best fit model (lines) and residual plots (lower panels) are shown. Titrations were performed at 25 °C, in 50 mM Tris-HCl buffer pH 7.6 for titrations with phosphate and in 50 mM Tris-HCl buffer pH 7.6 with 5 mM phosphate for titrations with formycin A, with the following initial enzyme concentration: 43.98 µM (brown), 144.87 µM (orange) and 699.90 µM (green) for titrations with phosphate, and 23.48 µM (brown), 69.28 µM (orange), 266.86 µM (green) for titrations with formycin A. Concentrations are calculated for the enzyme trimer, because the three-binding-site model was fitted as a global model. Parameters obtained are: K_d1_ = 16.52 µM, ΔH_1_ = -5.36 kcal/mol, K_d2_ = 154.24 µM, ΔH_2_ = -0.13 kcal/mol, K_d3_ = 79.67 µM, ΔH_3_ = -1.34 kcal/mol for titrations with phosphate and K_d1_ = 8.85 ± 1.91 µM, ΔH_1_ = -6.57 ± 0.12 kcal/mol, K_d2_ = 55.2 ± 19.7 µM, ΔH_2_ = -3.508 ± 2.993 kcal/mol, K_d3_ = 812.8 ± 1,924.5 µM, ΔH_3_ = -11.027 ± 24.350 kcal/mol for titrations with formycin A.

We have obtained some puzzling results not only regarding specific activity of the hexameric *E. coli* PNP. In the course of our long lasting project we have done also many titration experiments of this enzyme with various ligands in order to study details of complex formation of *E. coli* PNP with their substrates and inhibitors. Several detection techniques were used, fluorimetry, thermophoresis, circular dichroism and isothermal calorimetry. Examples of such experiments of *E. coli* PNP titrated with phosphate, and *E. coli* PNP-phosphate complex titrated with formycin A, utilizing the isothermal calorimetry as a detection method, are shown in Fig. 3. Protein samples used in these titrations were always electrophoretically pure, but had various specific activity, not always the highest observed for this enzyme, which means that in some samples part of the enzyme lost ability to conduct catalysis of inosine glycosidic bond cleavage. The concentration of a ‘catalytic’ fraction of the protein in such cases may be calculated as a ratio of the actual specific activity of the sample to the maximal specific activity of the enzyme vs. Ino ever observed, 117 U/mg^14^, multiplied by a ‘total’ protein concentration obtained from the UV spectrum taken before ligands were added.

Since some fraction of the protein used in titrations was not ‘catalytic’, in the analysis of the titration curves the concentration of the protein fraction ‘incompetent’ for ligand binding was always determined by introducing this parameter into the fitted model (as a local parameter for each titration curve, calculated in the Sedphat programme^28^, see “Materials and methods” Section for details). Calculation of the ‘total’ protein concentration from the UV spectrum allows to determine the ‘ligand-binding’ protein concentration, it means fraction actually involved in binding of the ligand, by simple subtraction the concentration of the ‘incompetent’ fraction from the ‘total’ protein concentration. The simple two-state model, in which the enzyme is either active or is not active, needs the concentration of ‘binding’ and ‘catalytic’ fractions to be the same. However, inspection of data shown in Table 1 clearly indicates that this is not the case for *E. coli* PNP. Concentration of the ‘binding’ fraction is always higher than that of the ‘catalytic’ fraction. This suggests that in the *E. coli* PNP aging process an intermediate or intermediates are present. In such an intermediate state enzyme molecules are able to bind ligands, but are not able to conduct catalysis, at least with inosine, which was the substrate used to calculate the ‘catalytic’ PNP fraction.

**Table 1:** Comparison of the total protein concentration, ligand-binding protein concentration and catalytically active protein concentration of several *E. coli* PNP samples.

	Titrations with phosphate		
	ITC experiment 1	ITC experiment 2	ITC experiment 3
Specific activity of the protein sample vs. Ino [U/mg]	66.22 ± 1.02	59.22 ± 2.13	39.36 ± 0.32
‘Total’ protein concentration ^a^ [µM]	43.98	144.87	699.90
‘Incompetent’ protein fraction^b^ [fraction of the total concentration]	0.099	0.229	0.136
‘Ligand-binding’ protein concentration^c^ [µM]	39.63	111.69	604.71
‘Catalytic’ protein concentration^d^ [µM]	24.85	73.33	235.45

	Titrations with formycin A		
	ITC experiment 4	ITC experiment 5	ITC experiment 6
Specific activity of the protein sample vs. Ino [U/mg]	29.16 ± 0.92	37.11 ± 0.57	76.69 ± 5.78
‘Total’ protein concentration^a^ [µM]	23.48	69.28	266.86
‘Incompetent’ protein fraction^b^ [fraction of total concentration]	0.0784	0.0664	0.0050
‘Ligand-binding’ protein concentration^c^ [µM]	21.64	64.68	265.53
‘Catalytic’ protein concentration^d^ [µM]	5.85	14.53	174.92

^a^Determined from the protein absorption spectrum with the extinction coefficient ε^1%^ = 2.7 mg^−1^ cm^− 1^^23^ and the molecular weight MW = 3 × 25,950 Da (calculated in ProtParam,^29^) (trimer). Concentration was calculated as a trimer concentration, as a three-binding-site model was fitted.

^b^Determined as a fitting parameter in the Sedphat programme^28^.

^c^Determined as ‘ligand-binding’=‘total’-‘incompetent’ concentrations.

^d^Determined as the ratio of the specific activity (vs. Ino) of the protein sample tested to the maximum activity of *E. coli* PNP, 117 U/mg^14^.

### Dissociation constants describing ligand binding change when specific activity of the enzyme changes

All these observations, coming from various experiments that basically had different goals than studies of the aging process of the *E. coli* PNP, drew our attention to the fact that this enzyme can exist in more than two states during aging, not only in the catalytically active and catalytically inactive states. Therefore, we decided to systematically monitor the time-dependent changes in the specific activity and ligand binding properties of this enzyme stored under different conditions. The samples tested had concentrations of about 4 µM for activity decay studies, and 7–25 µM for titrations with phosphate, and all were stored in the dark at 25 °C. The deactivation process was followed over the extended period of time in two ways: by performing a series of fluorescent titrations of the protein with phosphate to determine the ligand binding mechanism and dissociation constants, and by measuring the specific activity of the protein vs. two substrates, Ino and m^7^Guo, since a huge discrepancy was observed in the decrease of activity toward these two nucleosides, as indicated in the “Elusive specific activity of *E. coli* PNP” Section.

In the ligand-binding studies, the most important was to determine the mechanism and dissociation constants of the protein samples with various specific activities, from the highest possible to the almost completely vanished. Therefore, the activity of individual samples was monitored regularly, and when it reached a certain level, a series of titrations with phosphate was performed. Series done for the enzyme sample with a particular specific activity consisted of five titrations with different protein concentrations. The experiments were carried out as described in “Materials and methods” Section. All titrations were done exactly in the same way, so the only variable parameter was the specific activity of the studied *E. coli* PNP samples.

**Fig. 4:**
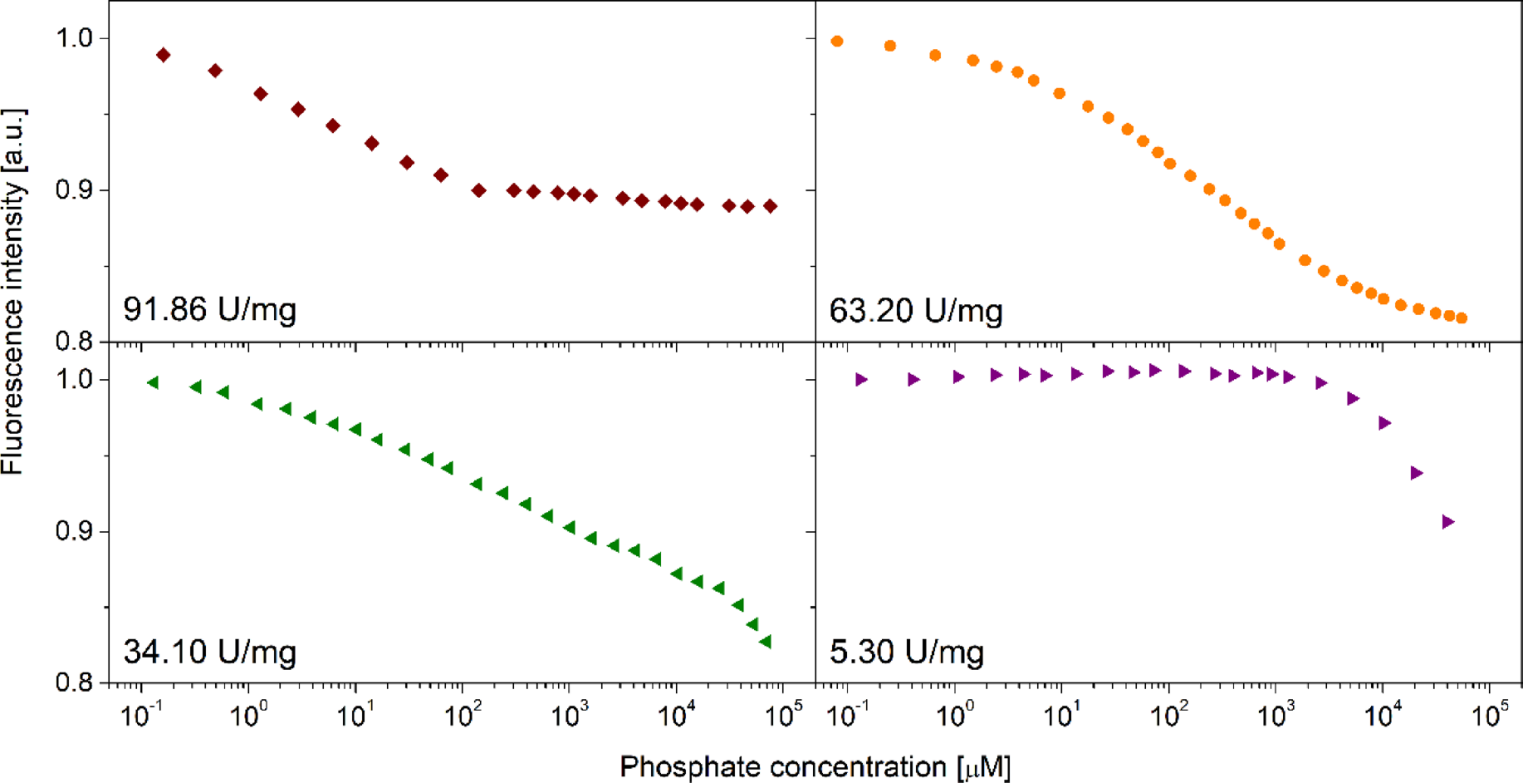
Titration curves of the *E coli* PNP with phosphate. The titrated enzyme had varying specific activities vs. Ino, as indicated in the individual panels. Data for just four enzyme specific activities is shown, but in total experiments were carried out for enzyme samples with fifteen different specific activities (see Supplement). Each experiment consisted of five titrations, each with a different protein concentration. Here only one curve is shown from each series of five titrations. The initial protein fluorescence, that was observed in titrations of the enzyme samples with different specific activities, was normalized to 1 to facilitate comparison of the shape of the titrations curves.

The data obtained (Fig. 4 depicts titration curves acquired for four exemplary enzyme samples with different specific activities) show that aging of *E. coli* PNP, leading to the decrease of the enzyme specific activity, has also a clear effect on the formation of the enzyme-phosphate ion complex, reflected in the shape of the titration curves. Visual inspection suggests that aging of the protein and gradual loss of the catalytic activity correlates with a weakening of phosphate binding, as for the enzyme samples with 34.14 U/mg and 5.29 U/mg saturation is not observed even for the highest phosphate concentration, 70 mM, used in the experiment (Fig. 4, lower panels).

Series of titrations obtained for each enzyme specific activity were analysed globally as described in “Materials and methods” Section. Three various models were taken into account, with one, two or three different dissociation constants of the ligand. The latter model is based on the crystallographic structures of the enzyme with phosphate (with or without nucleoside), where, as described in previous sections, two of the dimers are in the open-closed conformation of the active site, while the third dimer is in the open-open conformation. Therefore, the analysis included a model allowing for the possibility that the active sites in the open active site conformation belonging to the open-closed and open-open dimers may not be equivalent.

**Fig. 5:**
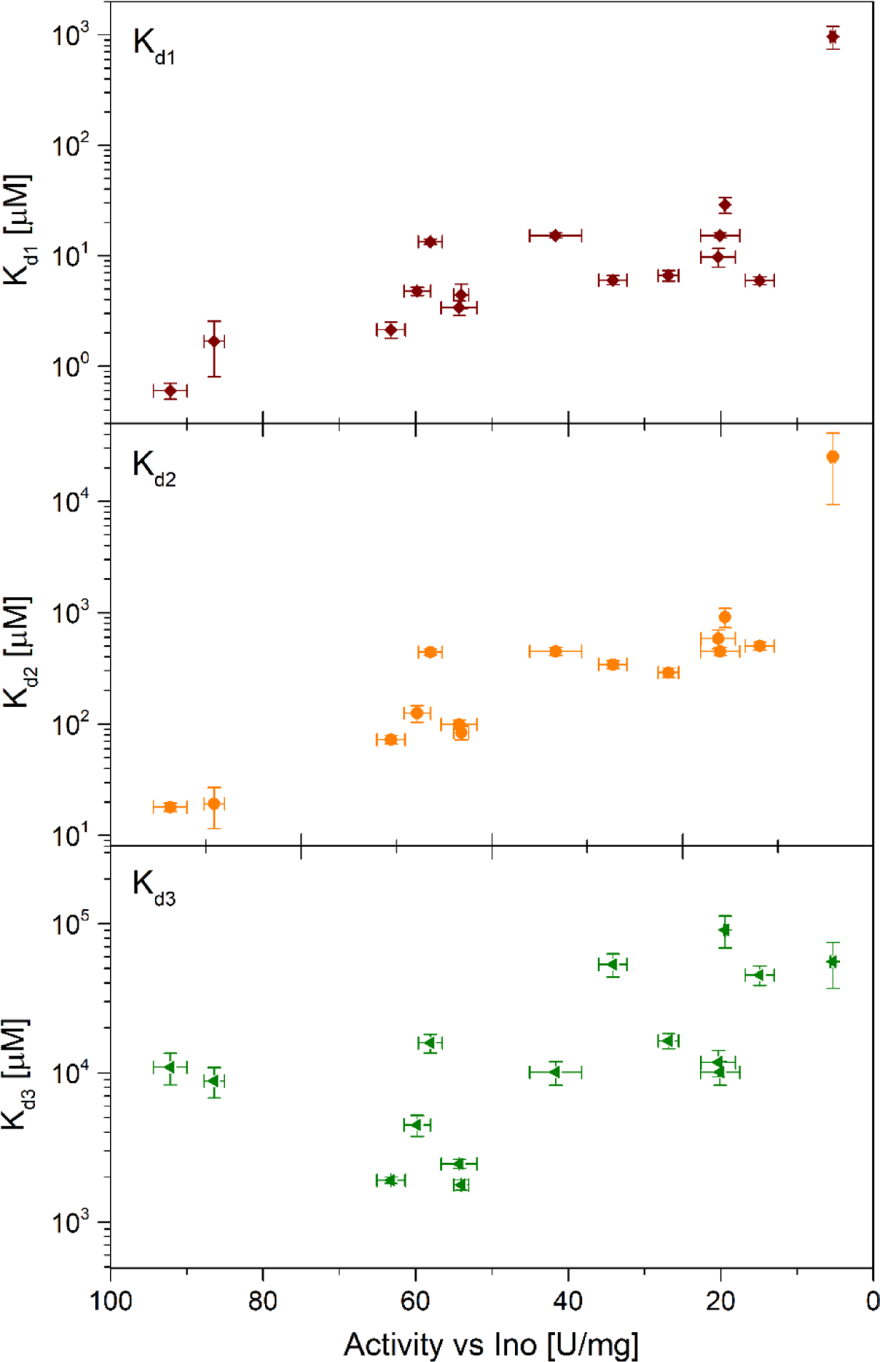
Dissociation constants describing interactions of the *E coli* PNP with phosphate as a function of the specific activity of the enzyme vs. inosine. Three-binding-site model was fitted to the titration data Examples of titration curves are depicted in Fig. 4.

Indeed, the discriminant analysis shows that the three-binding-site model best describes the observed titration curves. Values of the dissociation constants obtained, depicted as a function of the *E. coli* PNP specific activity, are shown in Fig. 5, and are also collected in the Supplement in a tabulated form (Table S2). The decrease in the specific activity of the enzyme is associated with a monotonic increase of dissociation constants K_d1_ and K_d2_ values (hence decrease in affinity to the ligand), whereas for the constant K_d3_ a small decrease is initially observed, and only later a monotonic increase occurs. These results demonstrate that the lower than the maximal specific activity of this enzyme does not simply indicate a lower concentration of the active protein in the sample. This supports the hypothesis that in the *E. coli* PNP aging process an intermediate or intermediates are present, which are able to bind ligands, albeit weaker than the fully active enzyme, but are not able to conduct catalysis of the natural substrate, inosine. This means that specific activity is the important parameter characterizing this protein, and that the results of experiments performed using different *E. coli* PNP samples can only be compared if the specific activities of the individual samples are similar.

### Decline of the specific activity vs. Ino and m^7^Guo of the aging *E. coli* PNP

**Fig. 6:**
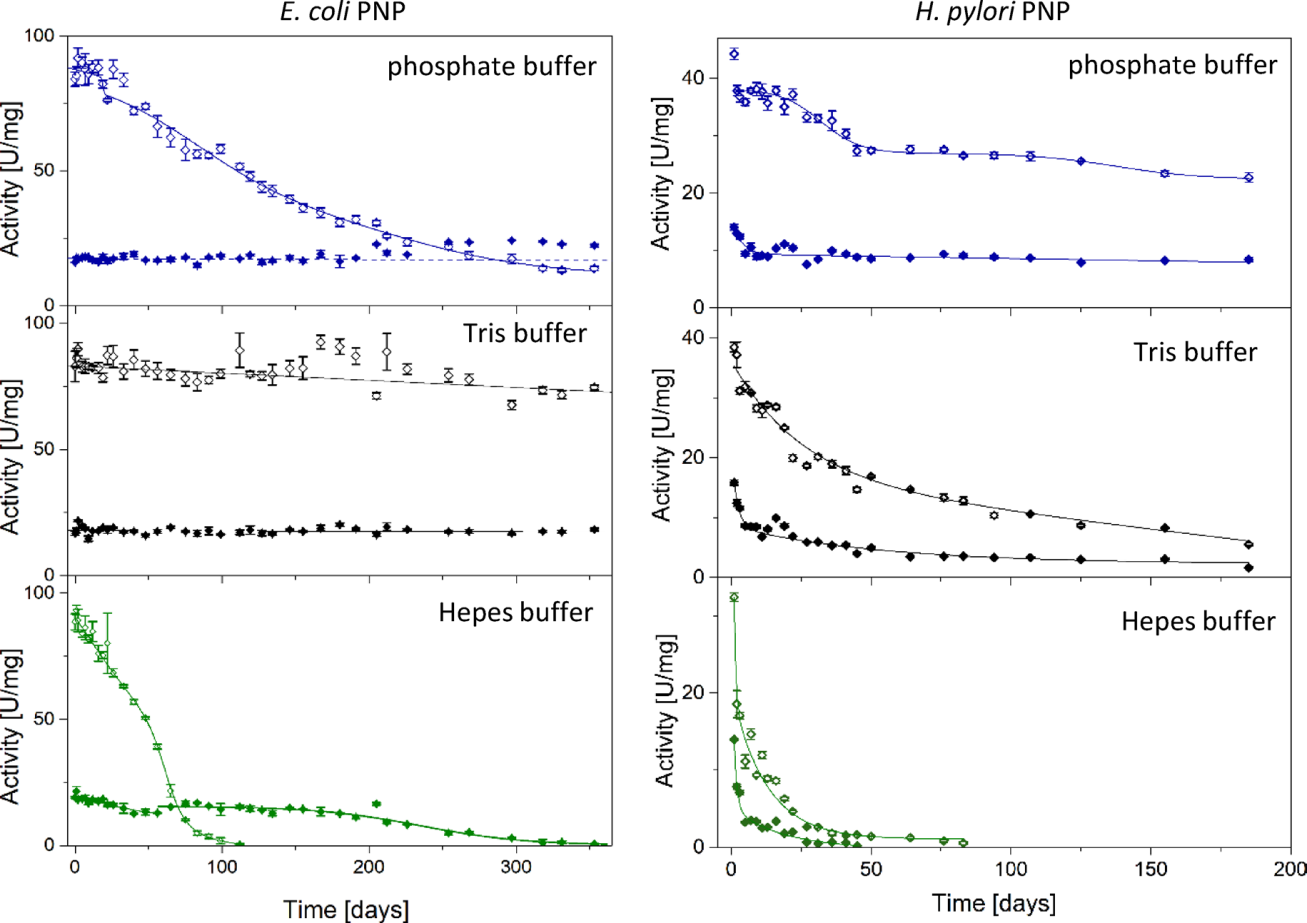
Decline of *E coli* PNP (left) and *H. pylori* PNP (right) specific activity vs. inosine (open symbols) and 7-methylguanosine (full symbols) in three various 50 mM buffers pH 7.6 at 25 °C.

In parallel with the measurements of the phosphate binding by the aging protein, experiments were performed to determine the profile describing the decline of the enzyme specific activity. To check whether this decline occurs at the same rate for different substrates, inosine and 7-methylguanosie, and in different buffers, the 50 mM pH 7.6 Tris-HCl, Hepes-NaOH and phosphate buffers were used. Phosphate was chosen as the enzyme substrate, known to stabilize the enzyme^18,30,31^, Hepes as a neutral buffer, having no influence on the enzyme activity, while Tris was also used because this molecule was found in the pentose binding-site in the crystal structure of some hexameric PNPs (e.g.^9,26^). To check whether the unusual molecular features and behaviour noticed for the *E. coli* PNP and described in previous sections also characterize other hexameric PNPs, we have conducted similar studies for the PNP from *Helicobacter pylori*. Enzymes from the purine salvage pathway, primarily PNP, have recently been identified as promising molecular targets in the design of new antibiotics to combat this dangerous human pathogen^6,9^. Therefore, properties of the PNP from *H. pylori* are important also from the medical point of view. The results obtained, describing the decline in the enzyme specific activity, are depicted in Fig. 6, on the left panels for the *E. coli* PNP and on the right panels for the *H. pylori* PNP.

The first striking observation is that the activity decreases in a different way versus two studied substrates, inosine and 7-methylguanosie, and in the extreme cases, that is for *E. coli* PNP vs. m^7^Guo in Tris and phosphate buffers, the activity does not decrease at all in the time period of the experiment, which is about a year. In most cases more than one phase is visible in the activity decline curves, suggesting the existence of intermediates. However, it is obvious that the activity decrease observed for both studied enzymes, from *E. coli* and from H. pylori, have different characteristic. The rate of decline of *H. pylori* PNP activity towards both substrates is more or less similar, and always specific activity vs. Ino is higher than that observed for m^7^Guo. By contrast, *E. coli* PNP losses activity vs. Ino much quicker than vs. m^7^Guo, resulting in time periods when the enzyme is more active vs. the latter substrate, although in the beginning activity vs. Ino is about 3-fold higher than vs. m^7^Guo (see Fig. 6).

The dose-response function, exponential decay, straight line or their combinations were fitted to the data (see Eqs. (2)–(6) in “Materials and methods” Section). Half-time values for the individual phases in the decay curves (and activity decay per day in the case of linear fragments) are depicted in Table 2. All parameters fitted to each of the decay curves are shown in Table S3 in the Supplement.

As expected, phosphate and Tris buffers, as enzyme ligands, stabilize *E. coli* PNP, and in these two buffers the enzyme loses activity much slower than in the Hepes buffer. However, this stabilization differs significantly when we compare the ability of the enzyme to catalyse the phosphorolysis of Ino with the ability to catalyse the phosphorolysis of m^7^Guo. In phosphate and Tris buffers, the activity vs. m^7^Guo is constant throughout the whole course of the one-year experiment (Fig. 6; Table 2), whereas the activity towards Ino decreases in both buffers, although much slower in Tris than in phosphate buffer. In Tris buffer over the period of observation, which is about one year, slow decay is observed that seems linear (0.028 ± 0.005 (U/mg)/day), while in the phosphate buffer three phases are observed.

The most noticeable and peculiar is the fact that decrease rates vs. both substrates in phosphate buffer differ so much that in these conditions activity profiles as a function of time cross each other. The enzyme that was about three times more active vs. Ino than vs. m^7^Guo at the beginning of the experiment, after about 250 days became more active vs. m^7^Guo than vs. Ino. But the most striking effect observed is that in the Hepes buffer, after about 100 days, *E. coli* PNP completely lost its activity vs. Ino, but for the following more than 200 days of the experiment was still active vs. m^7^Guo. It proves that our observations, described in previous sections, that the *E. coli* PNP after losing activity vs. natural substrate, Ino, is still able to catalyse phosphorolysis of the substrate resembling the transition state, m^7^Guo, were not artifacts.

At this point it should be recalled, that a quick phase in the m^7^Guo phosphorolysis was also observed (see Fig. 2) for the double mutant Asp204Ala/Arg217Ala of the *E. coli* PNP. Due to these two mutations the enzyme is unable to protonate the nucleoside substrate, resulting in activity towards natural substrates on the 0.1% level comparing with that exhibited by the wild type enzyme^18^. This suggests that, similarly as in the case of the wild type enzyme, the most active form of the double mutant is pretty unstable. It also may imply that a proper subunits cooperation is important not only for the synchronised closing and opening of active sites in order to protonate the substrate, but in the whole course of catalysis, as m^7^Guo protonation, hence also active site closing, is not necessary for the phosphorolysis of this substrate. It is further supported by the results of Štefanić et al.^32^, showing by the crystallographic snapshots, the nucleoside binding process by the double mutant Asp204Ala/Arg217Ala of the *E. coli* PNP. This study demonstrates that these two mutations led to the reverse nucleoside binding sequence. While in the partially occupied wild type enzyme nucleoside is found in the closed sites, in the double mutant open sites are occupied first.

**Table 2:** Time constants describing deactivation curves of *E. coli* PNP and H. pylori PNP versus Ino and m^7^Guo, in three 50 mM buffers (see Fig. 6), obtained as parameters of the fit of the linear, the exponential, the dose-response function or their combinations (see Eqs. (2)–(6) in “Materials and methods” Section). The values shown (except that for linear decay phases) should be considered as half-times of the individual phases observed in a decay being tested. All parameters fitted to each of the decay curves are shown in the Table S3 in the Supplement.

	Phosphate buffer pH 7.6	Tris buffer pH 7.6	Hepes buffer pH 7.6
*E. coli* PNP			
Ino	Three phase logistic (19.0 ± 1.6) days (81.1 ± 22.5) days (227.2 ± 121.2) days	Very slow decrease of activity 0.028 ± 0.005 (U/mg)/day	Two phase logistic (6.8 ± 1.9) days (61.5 ± 1.0 ) days
m^7^Guo	No decrease of activity 0.002 ± 0.004 (U/mg)/day	No decrease of activity 0.0014 ± 0.0021 (U/mg)/day	Two phase logistic* (14.1 ± 48.9) days (235.1 ± 6.8) days or Three phase logistic (26.8 ± 3.6) days (62.0 ± 3.5) days** (230.8 ± 5.8) days
*H. pylori* PNP			
Ino	Two phase logistic (31.9 ± 2.7) days (137.6 ± 20.8) days	One phase exponential + line (25.3 ± 4.7) days 0.056 ± 0.011 (U/mg)/day	Two phase exponential ~ 0.7 days*** (12.6 ± 1.6) days
m^7^Guo	One phase exponential + line ~ 3.3 days*** 0.008 ± 0.004 (U/mg)/day	Two phase exponential ~ 1.9 days*** ~ 53.5 days***	Two phase exponential ~ 1.0 days*** (17.9 ± 6.8) days

*Preferred model.

**Increase, i.e. positive h coefficient in a dose-response equation.

***Big errors.

Inactivation process of the *H. pylori* PNP does not resemble much that of *E. coli* PNP. The most active form of *H. pylori* PNP is relatively unstable, more unstable than that of *E. coli* PNP, regardless the substrate studied and conditions applied (Fig. 6; Table 2). In Hepes buffer, which does not bind with the enzyme, activity of the *H. pylori* PNP decreases exponentially and the half-life of the slower phase is about 12.6 and 17.9 days vs. Ino and m^7^Guo, respectively (Table 2). Initial activity vs. Ino is lower than in two other buffers, and we practically only partially see the first, very quick phase of the activity decay, with half-lives of about 0.7 day with Ino and 1.0 days with m^7^Guo as a substrate. Tris and phosphate buffer stabilize the enzyme, and in these conditions two phases in the activity decay curves are observed. When m^7^Guo is a substrate, in both buffers a quick initial phase (with a half-life 1.9 and 3.3 days in Tris and phosphate buffer, respectively) is followed by a slow activity decrease. In Tris buffer the half-life of this phase is 53.5 days, while in the phosphate buffer the decay is so slow (0.008 ± 0.004 (U/mg)/day) that over the observation period of about 200 days it seems linear. Activity of the *H. pylori* PNP vs. Ino also decays in two phases, but characteristic time constants are higher than when the substrate is m^7^Guo. It is about 30 days for the first, quicker phase. The second phase in phosphate buffer is so slow that activity change seems to be linear (0.056 ± 0.011 (U/mg)/day), while in Tris buffer half-time is about 138 days.

### Enzyme structure vs. activity decline and ligand binding ability

Although PNPs from *E. coli* and *H. pylori* have only 55% sequence similarity, their superimposed crystallographic structures are almost identical, including secondary structure elements (Fig. S2) and solvent accessible surface area, which is 141431.453 Å^2^ and 140848.656 Å^2^, for *E. coli* and *H. pylori* PNPs, respectively (calculated with PyMol). Comparison of the subunits with the open active site conformation from the apo structures of both enzymes, 1ecp (*E. coli* PNP)^12^ and 6f5a (*H. pylori* PNP)^33^ yields the RMSD of 0.82 Å. In both structures all subunits are in the open active site conformation. Moreover, the key amino acids forming the active sites of both proteins are identical: His4 and Arg43 come from the neighbouring subunit, Arg87, Arg24 form hydrogen bonds with phosphate while Glu181, Arg217, Gly20, Phe159 interact with a nucleoside. In both enzymes Asp204 and Arg217 are involved in catalysis. Closing of the active site by the segmentation of the H8 helix (see Fig. 1) is necessary to bring these two residues in the hydrogen bond distance, which is the first step in the mechanism of the glycosidic bond cleavage^14^. There is only one small difference: the active site residue Ser90 in *E. coli* PNP is replaced by Thr90 in *H. pylori* PNP. Despite these similarities, the observed activity decay curves observed for both enzymes differ markedly as shown in Fig. 6. First of all, phosphate stabilizes the *H. pylori* PNP the most, while for *E. coli* PNP this role is played by Tris molecule. Second, for *H. pylori* PNP the reversal of activity level vs. Ino and m^7^Guo is not observed, while this phenomenon occurs for *E. coli* in phosphate and Hepes buffers. Moreover, in the latter buffer *E. coli* enzyme adopts a state, in which is not active vs. Ino, while still its activity vs. m^7^Guo is close to its initial value (Fig. 6, lower left panel).

**Fig. 7:**
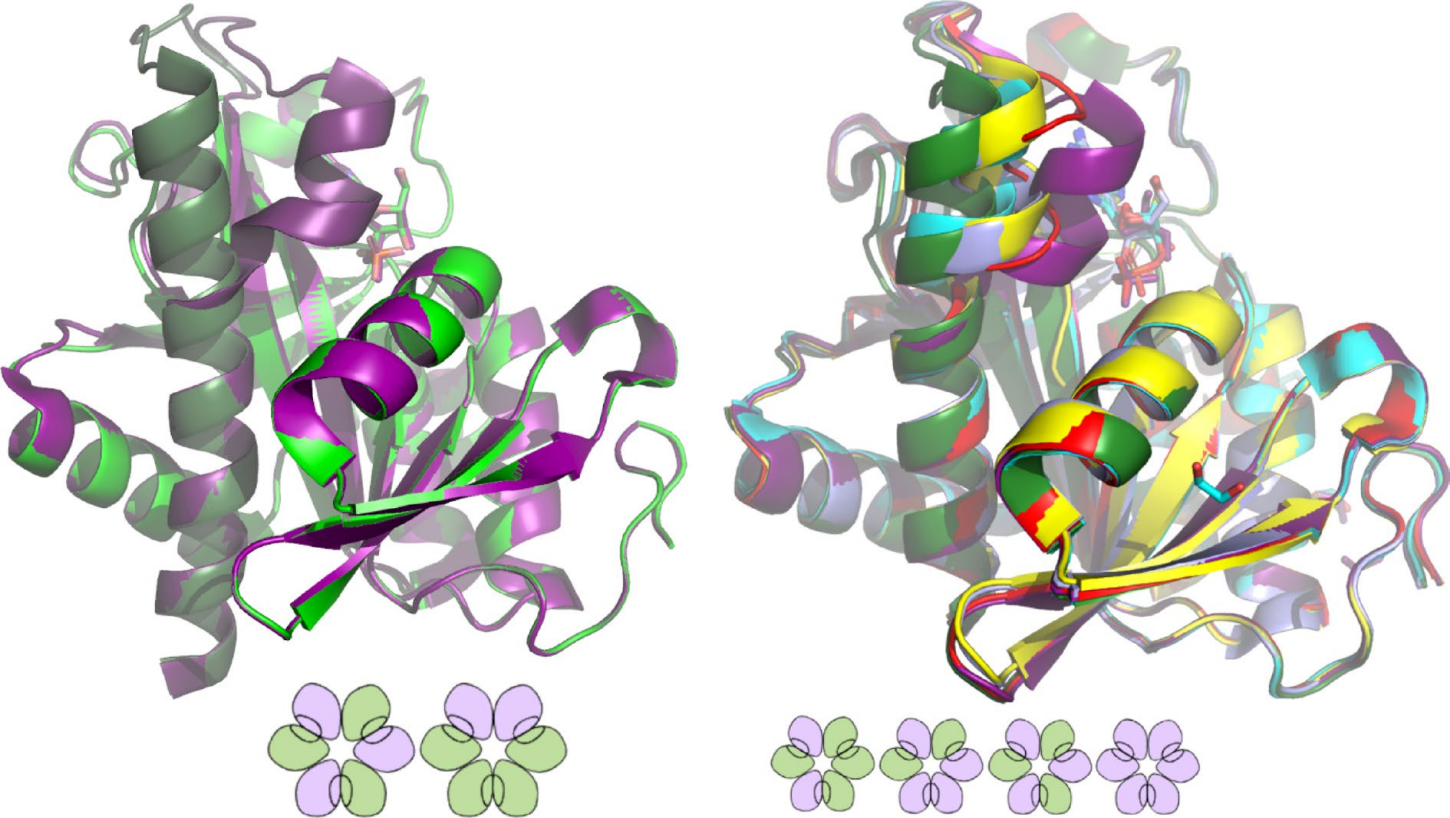
Upper panel: Aligned monomers of *E. coli* PNP structures (upper left panel) with phosphate and formycin, PDB 4ts9^32^, monomer C, green, in the open conformation of the active site, and monomer A, violet, in the closed conformation; and *H. pylori* PNP structures (upper right panel, all structures from Narczyk et al.^33^ with phosphate (PDB 5lu0, monomer F green, monomer E red, monomer B violet), with phosphate and formycin A (PDB 6f4x, monomer A cyan, monomer D yellow). Monomers of all available in PDB structures of each enzyme were aligned, and for each conformation of the N-terminal part of helix H8 only one was left visible for the clarity of the picture. Structure of the enzyme monomers in the open and closed active site conformations, in both *E. coli* PNP and *H. pylori* PNP, differ in principle only in the structure of the helix H8 and position of its N-terminal part. However, also differences in the solvent accessible surface areas are observed (see text). Lower panel: Scheme of two possible distributions of closed (lilac) and open (green) subunits observed in available structures in the hexamer of *E. coli* PNP (lower left panel) and four possible distributions observed in the *H. pylori* PNP (lower right panel).

If we focus on the conformational change observed in the structure of hexameric PNPs, which is triggered by binding of phosphate, and results in closing of the enzyme active site pocket by the N-terminus of the H8 helix, differences between both studied enzymes can be also seen. In all determined structures, active sites of *E. coli* PNP are observed only in two conformations, open (o) and closed (c) (Fig. 7, upper left panel). In contrast, for *H. pylori* PNP multiple positions of the N-terminus of the H8 helix are possible, hence active site may be also closed only partially (Fig. 7, upper right panel). Moreover, in the *E. coli* PNP hexamer, only two distributions of the hexamer subunits with the open and closed active site conformations are observed (Fig. 7 lower left panel): 3 open + 3 closed (alternating open and closed, three o-c dimers), and 2 closed + 4 open (two o-c dimers, one o-o dimer, closed subunits adjacent to each other). Solvent accessible surface area is 23530.932 Å^2^, 23599.285 Å^2^ and 23758.436 Å^2^ for subunits with the closed active site conformation from the o-c dimer, open active site conformation from the o-c dimer and the open active site conformation from the o-o dimer, respectively (PDB 4ts9).

In the *H. pylori* PNP hexamer as many as four different distributions occur (Fig. 7 lower right panel): 2 closed + 4 open (two o-c dimers, one o-o dimer, closed subunits not adjacent), 3 open + 3 closed (alternately open and closed, three o-c dimers), 4 closed + 2 open (one c-c dimer, two o-c dimers) and 6 closed. Solvent accessible surface areas is 23763.686 Å^2^ for the open active site conformation (PDB 5lu0, monomer F), 23771.770 Å^2^ for the closed active site conformation (PDB 5lu0, monomer B), while for the partially closed active site conformations values of 23707.824 Å^2^, 23721.648 Å^2^ and 23762.975 Å^2^ are observed (PDB 6f4x monomers A and D, and PDB 5lu0 monomer E, respectively). It is visible that, by contrast to the *E. coli* PNP, in the *H. pylori* PNP structure there is no correlation between these values and a degree of the active site closure.

**Fig. 8:**
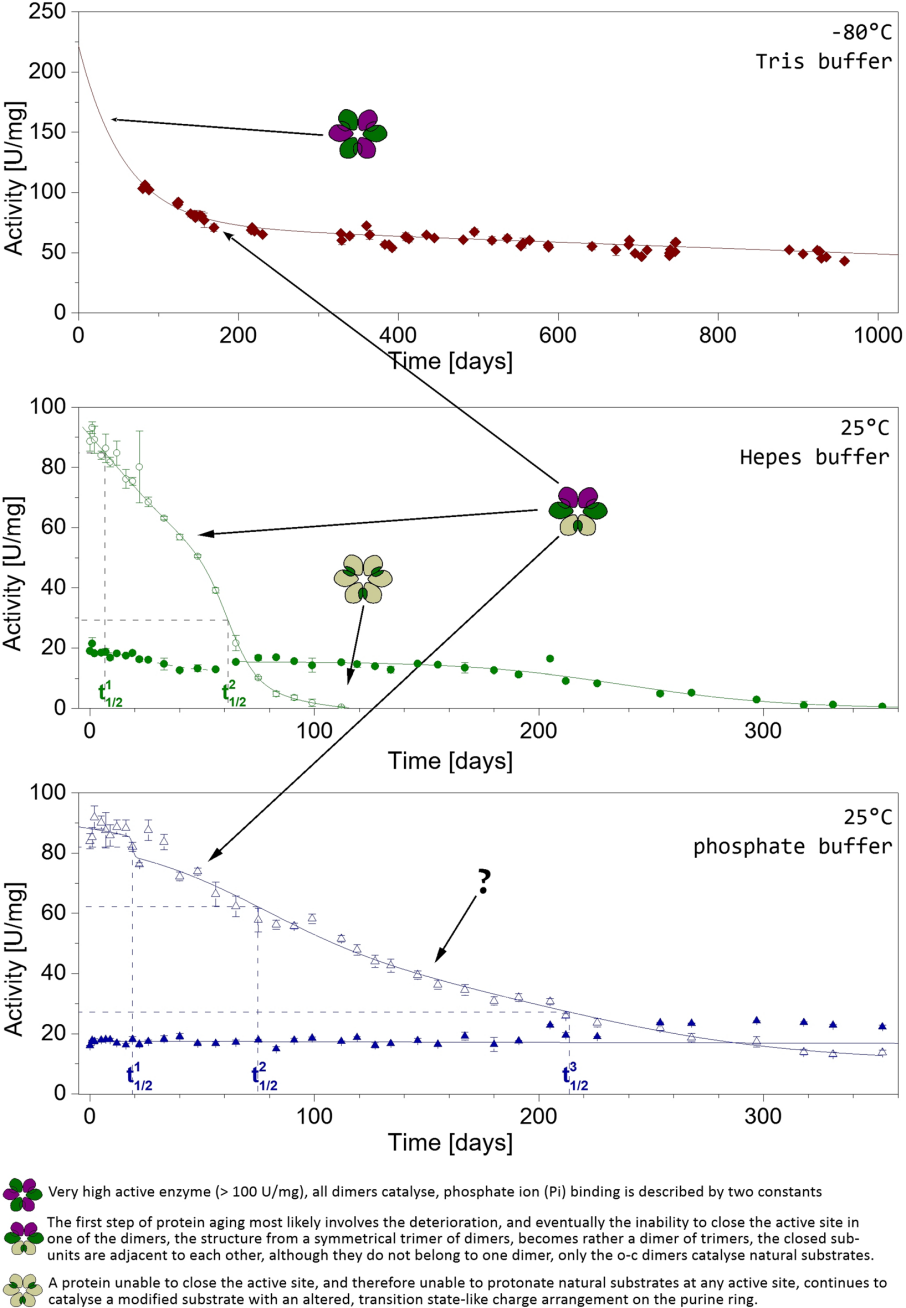
Postulated relations between the catalytic states of the *E. coli* PNP and its crystallographic forms. Decay curves of specific activity vs. Ino (empty dots) and vs. m^7^Guo (full dots). For two- and three-phase dose-response curves describing the decay of the protein activity vs. Ino, the vertical lines represent the half-times (t_1/2_) characterizing each stage and the horizontal lines the corresponding activity values. Protein subunits with the open active site conformation, but fully catalytically efficient hence able to stably close the active site (green); protein subunits with the closed conformation of the active site (violet); protein subunits with the open conformation of the active site and not able to stably close the active site, hence catalytically inactive towards natural substrates, however still capable of catalysing glycosidic bond cleavage of substrates such as m^7^Guo mimicking the transition-state of the reaction (olive).

Therefore, due to the several different distributions of the open and closed active site conformations within a *H. pylori* PNP hexamer, there is no clear connection between the specific structure of the enzyme and the observed phase of activity decay. The situation is different in the case of *E. coli* enzyme. This protein stored at -80 °C in 10 mM Tris buffer, which is a weak ligand, loses about 40% of its initial activity within three to four months and takes a more stable form, with an activity of about 70 U/mg, which inactivates more than ten times slower (Fig. 8, upper panel). The same change in the inactivation profile is observed for the enzyme stored at 25 °C (Fig. 8, middle and lower panels). This form of the protein is still capable of catalysing the phosphorolysis of natural substrates, so it can close the active site, but it is no longer fully catalytically efficient, so it probably structurally corresponds to the most commonly observed structure of this enzyme with two o-c dimers and one o-o dimer, i.e. with only two catalytically efficient dimers. Therefore, the most active form of the protein, with activity towards Ino above 100 U/mg, has all three dimers catalytically efficient. Indeed, in our previous studies such a structure was detected^14^ (PDB 1k9s), with all three dimers forming the protein molecule being the o-c dimers. The protein had an activity of 117 U/mg when crystallization was set. This is indeed the only reported structure of *E. coli* obtained for the fully or almost fully active form of this enzyme. The fully active form has probably even much higher activity as observed shortly after thawing the partially purified enzyme, as depicted on the Fig. 2. It is seen that this activity is between 200 and 300 U/mg, which is much more than just 3/2 of 70 U/mg characteristic for the enzyme with two o-c dimers and one o-o dimer. This may indicate that perfect cooperation of all six subunits results in synergistic effect, not just in adding activity of three dimers.

Next intermediate state suggested by the obtained data (Fig. 8) is probably a form of the protein, in which the enzyme is no longer able to catalyse phosphorolysis of natural substrates, but still catalyses the phosphorolysis of m^7^Guo due to its similarity to the transition state, as observed for the protein stored in 50 mM Hepes buffer pH 7.6. This means that none of the enzyme’s active sites can be stably closed. All subunits of the protein are in the open conformation of the active site, hence structurally the molecule more closely resembles the apo protein, or a complex with sulphate ions (PDB 3onv). Sulphate is the inhibitor, not a substrate of *E. coli* PNP, does not cause active site closing and its binding with the enzyme is characterized by just one dissociation constant. Finally, after one year of storage of *E. coli* PNP at 25 °C in 50 mM Hepes buffer pH 7.6, the enzyme becomes inactive also towards m^7^Guo. What is an overall structure of this completely catalytically inactive enzyme is not known so far. Trace activities of *E. coli* PNP dimers, as well as their poor stability revealed by molecular dynamics simulation^34^, suggest that the protein must remain in its hexameric form as long as it still has any reasonable catalytic properties.

Measurements of phosphate ion binding by the ageing protein, show a clear correlation between the catalytic activity of the protein and its affinity for the ligand. While for the dissociation constants K_d1_ and K_d2_ simple monotonic dependence on the specific activity of the enzyme towards Ino is observed, for the K_d3_ the observed dependence is more complicated, with an initial slight decrease, with a minimum for a protein having activity around 50 U/mg, and a subsequent monotonic increase. It is worth to mention here that in measurements of ligand binding by the enzyme, it is more difficult to detect individual protein transition states than in the activity decays profiles. If a mixture of protein species is present in the sample, which have different affinity to the ligand, the obtained dissociation constants for ligand binding are apparent constants resulting from averaging the values for each of the populations of molecular species in the sample. Nevertheless, the data obtained for phosphate binding of *E. coli* PNP are consistent with the relationship proposed above between the catalytic properties of the protein and its various crystallographic structures. The most active form of the protein is a trimer of dimers, which has only two populations of active sites, in open and closed conformations, with all dimers being the o-c ones. The phosphate ion binding should therefore be described by only two dissociation constants. Why then are the two series of titrations performed on the protein with the highest values of specific activity towards Ino (92.18 U/mg and 86.42 U/mg) described by three, and not two, dissociation constants? Because already at these protein activities, lower than maximal observed, some of the molecules in solution have one of the dimers in the o-o conformation, hence catalytically inoperative. The determined values of the K_d3_ constant are the apparent values for a mixture of the fully catalytically functional molecules (with three o-c dimers) and those with a ‘third’ dimer partially damaged. This ‘third’ dimer, being a dimer in the o-o conformation, is able to bind ligands, but is unable to catalyse phosphorolysis of natural substrates, so that a third constant appears in the binding curves as the specific activity of the protein decreases even slightly.

Further ageing of the protein finally leads to the loss of the ability of all three dimers of the molecule to close the active site. Such a protein is still able to bind ligands and catalyse the phosphorolysis of substrates mimicking the transition state (m^7^Guo). For a protein with activity towards Ino only 5 U/mg, the phosphate binding curve is virtually flat (Fig. 4), but the protein still catalyses the reaction of glycosidic bond cleavage of natural substrates with low efficiency, so it must bind substrates. This means that a protein with this activity behaves similarly to the Arg24Ala mutant, in which phosphate ion binding caused virtually no change in fluorescence intensity^35^.

**Fig. 9:**
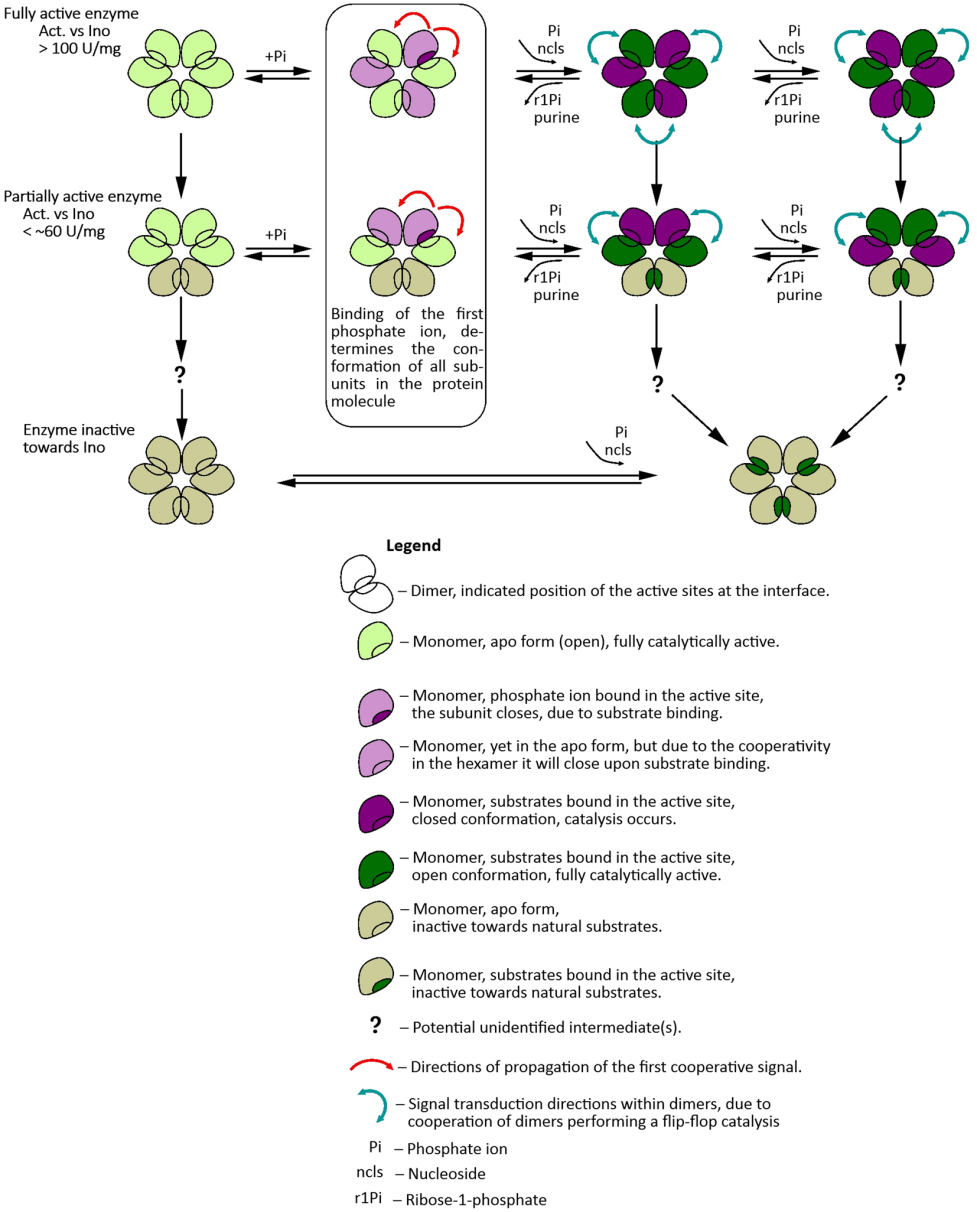
Stages in *E coli* PNP aging, from the fully active enzyme to the state where subunits are unable to close active sites and are therefore only active towards modified substrates such as m^7^Guo with a positive charge on the purine ring.

### Life cycle of *E. coli* PNP molecules

All the data presented above, together with the determined *E. coli* PNP three dimensional structures, allow us to propose a sequence of events occurring during the aging of the studied enzyme, i.e. during its “life cycle”. The fully active *E. coli* enzyme molecule, with the specific activity towards Ino much above 100 U/mg, has all subunits catalytically efficient (see Fig. 9). Each dimer carries out catalysis according to the flip-flop mechanism: subunits in each dimer alternately open and close active sites. The enzyme in this state acts as a real trimer of dimers, as catalysis in each dimer is synchronized with that of two other dimers. Pulsing in one rhythm all three dimers forming a hexamer close and open the active sites of one of their subunits. Moreover, subunits that are not adjacent to each other in the hexamer are closed (Fig. 9, upper row), so this alternating change of the state of a given subunit from the open active site to the closed active site applies to the entire hexamer. Such allosteric signal is probably dynamic in its nature as structure of ‘open’ and ‘closed’ subunits differ only in the position of the N-terminal part of helix H8, and slightly in the solvent accessible surface area. Subunits that close active sites probably stiffen, while cleavage of the glycosidic bond of the nucleoside substrate leads to the opening of the entrance to the active site pocket and loosening the structure again. This change is transmitted to the neighbouring subunit in the dimer and is a signal to close the active site and start catalysis. The cycles of stiffening and loosening of the structure of a given dimer most likely also affect neighbouring dimers. Thus, not only the subunits of a given dimer, but all three dimers in a hexamer cooperate closely with each other.

The aging of these efficiently cooperating subunits means that the proper transmission of signals between subunits is disturbed. The first intermediate observed in the aging process is a molecule in which one of the dimers in the hexamer becomes dysfunctional: the active site pocket of its subunits cannot be stably closed for the time necessary for the reaction to occur. The transmission of the cooperative signal for closing and opening active sites within this dimer is hampered or even absent, which results in impaired synchronization between all subunits in the hexamer. This leads to the formation of a new state of the molecule in which one of the dimers becomes catalytically inactive towards the natural substrates, while the reorganization takes place in the two remaining dimers. They are still able to catalyse the cleavage of the glycosidic bond of the natural substrates, but now the neighbouring subunits of these two dimers work in phase (see Fig. 9 middle row). This is the most abundant state, which molecules of *E. coli* PNP adopt, because was observed in almost all structures of this enzyme determined so far.

Is the next stage of aging of this protein a state in which only one dimer is catalytically active towards natural substrates? This would be the state marked with a question mark in Figs. 8 and 9. This cannot be excluded, but so far no structure showing such a state of the *E. coli* PNP is known. In the enzyme aging studies there is, however, the indication that such a stage could exist, as a three-step process of the activity loss towards Ino is observed for a protein stored in phosphate buffer, and may be also in Hepes buffer (Table 2).

The subsequent step in the ageing of *E. coli* PNP molecule is a state in which no subunit can close the active site. The enzyme is still able to bind ligands and catalyse glycosidic bond cleavage of modified substrates such as m^7^Guo, carrying the positive charge, hence not demanding active site closing and purine ring protonation. In this state, however, the enzyme is not capable to catalyse the phosphorolysis of natural substrates.

What mechanism causes the protein to inactivate in such a multistep way? Some light is shed on this subject by studies of dimers of *E. coli* PNP obtained by loosening interactions at the dimer-dimer interfaces^34^. Such dimers are not catalytically active. Without the support of neighbouring subunits, the dimer in solution is not stable, and a dynamic mixture of dimers and monomers is observed. However, such dimers anyway cannot be active, because molecular dynamics simulations showed also that although the dimer as a whole does not change its structure significantly when ‘removed’ from the hexamer, the big changes in position, relative to the initial crystallographic structure, involve the amino acids His4* and Arg43*, i.e. the amino acids from the neighbouring subunit that co-form the active site. Thus, the hexamer is needed for a dimer stability, hence also for the ability to perform catalysis.

The multitude distributions of open, closed, and partially closed active site conformations observed in crystallographic structures of *H. pylori* PNP, combined with no clear intermediates in the activity decay curves observed for this enzyme, may indicate that this molecule goes through multiple overlapping stages of inactivation, so that individual transition points cannot be distinguished. It could also mean that the cooperation between subunits in dimers of this enzyme is much weaker than in dimers of *E. coli* PNP (subunits work ‘more’ independently), or there is a very weak cooperation involving the whole hexamer and therefore the distribution of open and closed subunits is random or almost random in the hexamer. In line with this difference between both enzymes, is the absolute value of their specific activity vs. natural substrates, whose catalysis requires the closure of the active site. Namely, in the case of Ino, it clearly exceeds 100 U/mg for *E. coli* PNP, while it is only about 40 mg/ml for *H. pylori* PNP.

With that said, like any product of evolution, the protein is not perfect. Biological evolution is not directed towards finding perfect solutions, but through a process of natural selection, in which the worst solutions are eliminated from the pool of emerging solutions. If one catalytically inactive dimer in the hexameric PNP does not significantly reduce bacterial survival, then the gene encoding such a protein would not be subject to selection pressure. The ageing of the protein would therefore be related to the imperfection in functioning of the hexamer as a whole molecule. These results prompt one more question. What is the relation between the observed aging of the protein in vitro with that what happens in the cell? The half-life of PNP in bacterial cell is not known. Perhaps it is so short that in vivo a state of the reduced activity, characterised by a single inactive dimer, is not observed at all. It is more so probable, that interactions of PNP with other proteins in the crowded space of the cell stabilise the enzyme, and the observed stages in aging of this protein are observed only while the purified protein is present in solution containing only buffer molecules.

## Conclusions

Hexameric *E. coli* purine nucleoside phosphorylase, which is built as a trimer of dimers, does not inactivate in one step, from the fully active to the inactive enzyme. During the process of inactivation this protein passes the intermediate states with various catalytic and ligand binding abilities. The fully active state, in which all three dimers in a synchronised way close and open one of their subunits, is rather unstable. Only once such enzyme structure was captured. In this state ligand binding is characterised by two dissociation constants, describing binding to the active sites in the closed and open conformations, respectively. By contrast, pretty stable is the intermediate state of *E. coli* PNP with one catalytically inactive dimer, resulting from the fact that this dimer is unable to close the active site of its subunits. It is still able to bind ligands, but with dissociation constants that differ from that characterizing ligand binding by the subunits in the open active site conformation form of the dimer that is still catalytically active. Therefore, three-binding-site model, with three different dissociation constants, is necessary to describe ligand binding of the enzyme in this state. This intermediate, which has two closed-open dimers, and one open-open dimer, is the structure of *E. coli* PNP mostly observed. Next intermediate, with only one dimer catalytically active was not captured up to now, but there is an indication that is exists because three-step process of activity loss towards natural substrate, inosine, is observed for this enzyme. In the following state, when none of the dimers is able to close active site of its subunits, the enzyme is not capable to catalyse reaction with natural substrates, but is still able to bind ligands and is catalytically active towards 7-methylguanosine. This is because this substrate resembles the transition state of the PNP-catalysed reaction due to positive charge on the purine ring, hence protonation of 7-methylguanosine by the enzyme, resulting from closing of the active site, is not necessary.

Such well-defined intermediate states are not observed in the process of *H. pylori* purine nucleoside inactivation. Structures of this enzyme with various distribution of the open, closed and partially closed active sites of the subunits are observed, as well as various positions of the broken N-terminal part of H8 helix, including the fact that the most active *H. pylori* PNP form is not characterized by the three open-closed dimers with every second subunit closed. This lack of perfect synchronisation of dimers work may be responsible for the fact that the maximum specific activity towards inosine for *H. pylori* PNP is about 40 U/mg, while for *E. coli* PNP is well above 100 U/mg. It remains the open question if the molecular crowding in the cell environment stabilizes the fully active form of both enzymes.

## Materials and methods

### Enzymes and other chemical reagents

The source of the *E. coli* PNP in the first years of the project was a kind gift of Prof. Thomas Krenitsky and Dr. George Koszalka from the Wellcome Research Laboratories (Research Triangle Park, NC, USA). First we have got a purified enzyme with activity of 117 U/mg, followed by a huge amount of the partially purified with a declared activity of about 56 U/mg. The latter one was purified to homogeneity in one step by the affinity chromatography with the Sepharose CL-6B activated with the inhibitor of the enzyme, formycin B or formycin A, as described in our paper from that period^23^. In the further period, until now, the PNP from *E. coli* used in our studies has been a recombinant protein obtained by overexpression in *E. coli* cells^36^ and purified either by the ion-exchange and size-exclusion chromatographic steps^18^ or with the aid of the mentioned above affinity chromatography as described in Bzowska et al.^23^. PNP from *H. pylori* used in this project was a recombinant protein from the 26695 *H. pylori* strain obtained by overexpression in *E. coli* cells and purified as described previously^33^ using also the same affinity column as that utilized for *E. coli* PNP. For specific activity assays and for all other biophysical studies, enzyme concentration was determined from the UV spectra using the following extinction coefficients at 278 nm for a 1% solutions: ε^1%^ = 5.19 mg^−1^cm^− 1^ for PNP from *H. pylori* and ε^1%^ = 2.7 mg^−1^cm^− 1^ for PNP from *E. coli*. For *H. pylori* PNP it is based on the amino acid sequence (ProtParam,^29^) while for *E. coli* it is the experimentally determined value^23^. Taking molecular weight of the *E. coli* enzyme subunit MW = 25,950 Da, calculated in ProtParam for the *E. coli* deoD coded enzyme (P0ABP8), it corresponds to 7000 M^−1^cm^− 1^. It should be noted that this value slightly differs from the extinction coefficient calculated from the sequence^29^, which is 0.345 for a 0.1% solution corresponding to 8940 M^−1^cm^− 1^. If not otherwise indicated, the enzyme concentration is expressed in monomers.

The basic chemical reagents like buffers (Tris, phosphates) and other salts were products of Roth (Carl Roth GmbH and Co., Karlsruhe, Germany). The Hepes buffer was purchased from Sigma (Sigma-Aldrich, Saint Louis, Missouri, USA), as it contained lower concentrations of phosphate ions than the same buffer provided by other suppliers. Inosine and xanthine oxidase from butter milk were purchased also from Sigma, 7-methylguanosine was synthesised from guanosine using the method of Jones and Robins^37^ by Professor Tsutomu Yokomatsu (Tokyo University of Pharmacy and Life Science, School of Pharmacy). Phosphate-free HCl and NaOH, to adjust buffers pH, were from Fluka (Fluka, Honeywell Research Chemicals). Formycins A and B were first purchased from ICN Biochemicals (Irvine, California, USA), then, when this company stopped producing this item, from Berry & Associates (Dexter, Michigan, USA).

As the phosphate ion is one of the substrates of PNP, its content was controlled in all solutions used during the experiments. The colorimetric method of Ames^38^ was used. The residual concentration of phosphate ions was as follows: in 50 mM Tris buffer, the concentration of phosphate was barely detectable, namely in the initial 1 M solution it was 0.8 µM, while in the 50 mM Hepes buffer it was at the level of 1.2 µM.

### Measurements of the PNP activity

Specific activity of PNP vs. inosine and 7-methylguanosine was measured using spectrophotometric assays. In the case of inosine, the standard xanthine oxidase (Xox) conjugated reaction was used (λ_obs_ = 300 nm, Δε = 9600 M^−1^cm^− 1^)^11,24^, while in the case of m^7^Guo, the substrate concentration decrease was directly observed (λ_obs_ = 260 nm, Δε = -4600 M^−1^cm^− 1^)^20^. Measurements of specific activity of *E. coli* and *H. pylori* PNPs were carried out using various UV-VIS spectrophotometers, mainly a Cary100Bio apparatus (Agilent Technologies), at 25 °C, in 50 mM phosphate buffer pH 7.0 as a PNP substrate, and in a saturating concentration of the second substrate, nucleoside (0.5 mM and 1 mM of Ino, respectively for *E. coli* and *H. pylori* PNPs, and 200 µM of m^7^Guo). Cuvettes with the optical path length ensuring that the absorbance did not exceeded 1 were used, that is 1 cm with Ino as a substrate and 0.5 cm with m^7^Guo. Activity values were calculated as a mean of three to five measurements, with a mean standard deviation as an error. With the exception of samples whose deactivation occurred on a scale of minutes (see Section “Elusive specific activity of *E. coli* PNP”), this error never exceeded 5%.

### ITC titrations

Calorimetric titrations were conducted on ITC200 microcalorimeter (Malvern, UK). Protein solution for each experiment was dialysed against proper buffer, 50 mM Tris-HCl buffer pH 7.6 or 50 mM Tris-HCl with 5 mM phosphate pH 7.6, for titrations with phosphate and formycin A, respectively. Ligands were dissolved in buffer in which protein was dialysed. All samples were filtered through 0.22 μm filters and degassed prior ITC titrations. All experiments were carried out at 25 °C. A series of three titrations were performed for each ligand under study, with the protein concentration in the range of 40–700 µM, and 20–270 µM for titrations with phosphate and formycin A, respectively. Ligands samples were 8–90 and 11–70 times more concentrated than the protein sample to achieve a diverse ligand/protein molar ratios during titration, and thus better explore binding curves^39^. Raw thermograms were analysed with the NITPIC program^40^. Obtained titration curves were analysed globally with the ITCsy/Sedphat program^28,41^, which includes explicitly the parameter ‘incompetent fraction’ of the receptor, which means fraction of the protein not involved in the complex formation. Fitting of appropriate models were performed with total protein concentrations, that were determined from the UV absorption spectra, as initial parameters values, with incompetent fraction parameters, equal zero as the initial value, and used as variable parameter of the fit. The discriminant analysis was performed as described previously^21^.

### Fluorimetric titrations with phosphate of the aging *E. coli* PNP

Fluorescence measurements were conducted on a Perkin LS55 (Perkin Elmer, MA, USA) and a Tau-3 Fluorologist (Jobin Yvon Inc.) spectrofluorimeters. Semi-micro cuvettes were used, with a path length for the excitation of 2 mm, and for the emission − 5 mm (which are a half of the cuvette dimensions). A continuous stirring was used during titrations. All measurements were performed at 25 °C, in 50 mM Tris-HCl buffer pH 7.6. The specific activity vs. Ino of the aging *E. coli* PNP samples was monitored regularly, as described in the previous section, and when it reached a specific, desired level, a series of titrations with phosphate was performed. Each series consisting of five titrations performed at different protein concentrations. Enzyme concentration (as a monomer) was in the range of 0.5–2.5 µM. Protein specific activity was measured before each experiment, on a sample prepared for each titration, and then again after completing each titration, to control the condition of the protein during the course of the whole titration.

For each chosen specific activity of the aging protein, a series consisted of 3–5 titrations was done. Each series was analysed globally using the program DYNAFIT^42^, and three different binding models were taken into account, namely with one, two, and three binding sites. The best model was chosen on the basis of a discriminant analysis as described previously^21^. Briefly, titration series were analysed globally, always starting from the simplest, one-binding-site model. If the residual plots, judged visually and by Runs of Sign Test/Wald-Wolfowitz Runs Test (WWtest,^43^), suggested that one-binding-site model does not describe data well, the two-binding-site model was tested. Only if the analysis of the residual plots for the two-binding-site model, judged by the WWtest, suggested that still more complex model should be tested, the three-binding-site model was fitted. For all tested models the Akaike information criterion^44^ was also calculated. Overall, based on both criteria used, the three-binding-site model was found to best describe the experimental data for each series of titrations. Slightly problematic are the results obtained from the titrations performed on the protein with an activity of 19.46 U/mg and lower, because the third constant is close to the highest ligand concentration (70 mM) or even outside this range (Table S2 in the Supplement). Moreover, for the series obtained for the protein with the lowest activity of 5.29 U/mg, the Akaike criterion indicates the two-binding-site model as better, however the analysis of the residual plots points to the three-binding-site model as the best, although the errors of the fitted parameters are big. Therefore, Table 2 depicts parameters obtained for the three-binding-site model.

The models analysed were fitted to the raw data, while the graphs presented in this paper were in many cases prepared from the normalized data to allow for better visual comparison of titrations performed under different conditions.

### *E. coli* and *H. pylori* PNPs inactivation under forced, but mild conditions and analysis of the aging data

Three samples of *E. coli* PNP with a concentration of 4.06 µM were prepared in three different buffers, 50 mM pH 7.6: Tris-HCl, Hepes-NaOH and phosphate buffer. Samples were stored in the dark at 25 °C for about a year. At specific time intervals small volume from each sample was withdrawn and used for the measurements of activity towards two substrates, Ino and m^7^Guo, using the spectrophotometric assays as described in the Section “Measurements of the PNP activity”. At the beginning such activity was measured every day for each aging sample, then less and less frequently depending on the observed rate of decline in the specific activity. Similar experiment, lasting about six months, was done for the *H. pylori* PNP, and the concentration of the three aging samples prepared in the mentioned above buffers was 4.05 µM.

Data, i.e. A(t), specific activity time dependence of PNP activity towards Ino or m^7^Guo, was analysed using the program Origin (OriginPro, Version 8, OriginLab Corporation, Northampton, MA, USA). The linear, exponential, the dose-response functions or their combinations were fitted, with weighting, to the aging enzyme activity data. The strait line describes most probably also an exponential process, which however was followed too shortly to notice the curvature The following equations were used:

2 $$A\left( t \right) = kt + A_{0}$$

3 $$A\left( t \right) = A_{1} \exp ^{{\left( { - t/t_{1} } \right)}} + A_{2} \exp ^{{\left( { - t/t_{2} } \right)}} + A_{\infty }$$

4 $$A\left( t \right) = A_{1} \exp ^{{\left( { - t/t_{1} } \right)}} + kt + A_{2}$$

As a dose-response sigmoidal dependence, a generalized logistic function (called also Richards’s function^45^) was used:

5 $$A\left( t \right) = A_{\infty } + \left( {A_{0} - A_{\infty } } \right)\frac{1}{{\left( {1 + 10^{{\left( {t_{1} - t} \right)h}} } \right)}}$$

where A_0_ is the initial activity, A_∞_ is the residual activity, t_1_ is a time constant characterizing course of the activity change (time point in which activity drops to 50% of the A_0_- A_∞_ difference, (half-time)), while h is characterizing steepness of the curve, and is negative for the activity decreasing over time. In some conditions enzyme inactivation was found to proceed via intermediates, so in the most complicated cases a sum of three logistic functions were used, as follows:

6 $$A\left( t \right) = A_{\infty } + \left( {A_{0} - A_{\infty } } \right)\left[ {\frac{p}{{\left( {1 + 10^{{\left( {t_{1} - t} \right)h_{1} }} } \right)}} + ~\frac{q}{{\left( {1 + 10^{{\left( {t_{2} - t} \right)h_{2} }} } \right)}}~\frac{{1 - p - q}}{{\left( {1 + 10^{{\left( {t_{3} - t} \right)h_{3} }} } \right)}}} \right]$$

where p, q and 1-p-q are parameters characterizing the fraction of the total decay that occurs with the half-time t_1_, t_2_ and t_3_, respectively. When two phases described by the sigmoidal dose-response function were observed in the decay process, the parameter q = 0.

Experimental error of each point is calculated as standard deviation of the arithmetic average (StDev), and this error is used by the Origin program in the fitting procedure to calculate the weight (w), defined as:

7 $$w = \frac{1}{{StDev^{2} }}$$

Weighting is particularly important during fitting, where the standard deviation of the points in the series is significantly different, because it reduces the influence of points with large errors. As can be seen in Fig. 8 for the phosphate buffer around day 20–30, at this stage of the curve there are two points with large errors compared to the errors of the other points. The introduction of weights means that, although they are included in the analysis, they have little impact on the final result.

## Supplementary Information

Below is the link to the electronic supplementary material.

Supplementary Material 1

## Data Availability

The materials described in the manuscript, including all relevant raw data, will be made freely available upon a reasonable request to any researcher who wishes to use them for non-commercial purposes, sent to the corresponding author.

## Abbreviations

PNP: Purine nucleoside phosphorylase
o-c dimer: Enzyme dimer with one active site in the open conformation and one in the closed conformation
o-o dimer: Enzyme dimer with both active sites in the open conformation
Ino: Inosine
m^7^Guo: 7-methylguanosine
K_d_: Dissociation constant
K_i_: Inhibition constant
K_m_: Michaelis constant
V_max_: Maximal velocity

## References

[1] Bzowska, A., Kulikowska, E. & Shugar, D. Purine nucleoside phosphorylases: Properties, functions, and clinical aspects. *Pharmacol. Ther.* **88**, 349–425. https://doi.org/10.1016/s0163-7258(00)00097-8 (2000). 11337031 10.1016/s0163-7258(00)00097-8

[2] Giblett, E. R., Ammann, A. J., Wara, D. W., Sandman, R. & Diamond, L. K. Nucleoside-phosphorylase deficiency in a child with severely defective T-cell immunity and normal B-cell immunity. *Lancet* **1**, 1010–1013. https://doi.org/10.1016/s0140-6736(75)91950-9 (1975). 48676 10.1016/s0140-6736(75)91950-9

[3] Makita, S., Maeshima, A. M., Maruyama, D., Izutsu, K. & Tobinai, K. Forodesine in the treatment of relapsed/refractory peripheral T-cell lymphoma: An evidence-based review. *OncoTargets Ther*. **11**, 2287–2293. 10.2147/OTT.S140756 (2018). 29719411 10.2147/OTT.S140756 PMC5916385

[4] Bennett, E. M. et al. Designer gene therapy using an *Escherichia coli* purine nucleoside phosphorylase/prodrug system. *Chem. Biol.* **10**, 1173–1181. 10.1016/j.chembiol.2003.11.008 (2003). 14700625 10.1016/j.chembiol.2003.11.008

[5] Hassan, A. E. A., Abou-Elkhair, R. A. I., Parker, W. B., Allan, P. W. & Secrist III, J. A. 6-Methylpurine derived sugar modified nucleosides: Synthesis and in vivo antitumor activity in D54 tumor expressing M64V-*Escherichia coli* purine nucleoside phosphorylase. *Eur. J. Med. Chem.* **108**, 616–622. 10.1016/j.ejmech.2015.12.029 (2016). 26724729 10.1016/j.ejmech.2015.12.029

[6] Liechti, G. & Goldberg, J. B. Helicobacter pylori relies primarily on the purine salvage pathway for purine nucleotide biosynthesis. *J. Bacteriol.* **194**, 839–854. 10.1128/JB.05757-11 (2012). 22194455 10.1128/JB.05757-11 PMC3272961

[7] Madrid, D. C., Ting, L. M., Waller, K. L., Schramm, V. L. & Kim, K. *Plasmodium falciparum* purine nucleoside phosphorylase is critical for viability of malaria parasites. *J. Biol. Chem.* **283**, 35899–35907. 10.1074/jbc.M807218200 (2008). 18957439 10.1074/jbc.M807218200 PMC2602904

[8] Devi, K., Chandra, A., Chaudhuri, S. & Goel, V. K. Novel dipeptide inhibitors of PfPNP: In-silico identification of promising new antimalarials. *Chem. Biodivers.* **22**, e202401668. 10.1002/cbdv.202401668 (2024). 39345161 10.1002/cbdv.202401668

[9] Narczyk, M. et al. Interactions of 2,6-substituted purines with purine nucleoside phosphorylase from *Helicobacter pylori* in solution and in the crystal, and the effects of these compounds on cell cultures of this bacterium. *J. Enzyme Inhib. Med. Chem.* **37**, 1083–1097. 10.1080/14756366.2022.2061965 (2022). 35437103 10.1080/14756366.2022.2061965 PMC9037209

[10] Jensen, K. F. Purine nucleoside phosphorylase from *Salmonella typhimurium* and *Escherichia coli* Initial velocity kinetics, ligand binding and reaction mechanism. *Eur. J. Biochem.* **67**, 377–386. 10.1111/j.1432-1033.1976.tb10031.x (1976). 10.1111/j.1432-1033.1976.tb10031.x 813997

[11] Stoeckler, J. D., Agarwal, R. P., Agarwal, K. C. & Parks Jr, R. E. Purine nucleoside phosphorylase from human erythrocytes. *Methods Enzymol.* **51**, 530–538. 10.1021/bi00595a014 (1978). 99639 10.1016/s0076-6879(78)51074-4

[12] Mao, C., Cook, W. J., Zhou, M., Koszalka, G. W., Krenitsky, T. A. & Ealick, S.E. The crystal structure of *Escherichia coli* purine nucleoside phosphorylase: A comparison with the human enzyme reveals a conserved topology. *Structure* **5**, 1373–1383. https://doi.org/10.1016/s0969-2126(97)00287-6 (1997). 9351810 10.1016/s0969-2126(97)00287-6

[13] Koellner, G., Luič, M., Shugar, D., Saenger, W. & Bzowska, A. Crystal structure of the ternary complex of *E. coli* purine nucleoside phosphorylase with formycin B, a structural analogue of the substrate inosine, and phosphate (sulphate) at 2.1 Å resolution. *J. Mol. Biol.* **280**, 153–166. 10.1006/jmbi.1998.1799 (1998). 9653038 10.1006/jmbi.1998.1799

[14] Koellner, G. et al. Open and closed conformation of the *E. coli* purine nucleoside phosphorylase active center and implications for the catalytic mechanism. *J. Mol. Biol.* **315**, 351–371. 10.1006/jmbi.2001.5211 (2002). 11786017 10.1006/jmbi.2001.5211

[15] Bennett, E. M., Li, C., Allan, P. W., Parker, W. B. & Ealick, S. E. Structural basis for substrate specificity of *Escherichia coli* purine nucleoside phosphorylase. *J. Biol. Chem.* **278**, 47110–47118. 10.1074/jbc.M304622200 (2003). 12937174 10.1074/jbc.M304622200

[16] Štefanić, Z. et al. New phosphate binding sites in the crystal structure of *Escherichia coli* purine nucleoside phosphorylase complexed with phosphate and formycin A. *FEBS Lett.* **586**, 967–971.. 10.1016/j.febslet.2012.02.039 (2012) 22569248 10.1016/j.febslet.2012.02.039

[17] Timofeev, V. I., Abramchik, Y. A., Esipov, R. S. & Kuranova, I. P. Crystal structure of *E. coli* purine nucleoside phosphorylase with acycloguanosine. *Struct. Biol. Cryst. Commun.* **74**, 402–409. https://doi.org/10.1107/S2053230X18008087 (2018). 10.1107/S2053230X18008087 PMC6038453 29969103

[18] Mikleušević, G. et al. Validation of the catalytic mechanism of *Escherichia coli* purine nucleoside phosphorylase by structural and kinetic studies. *Biochimie* **93**, 1610–1622. 10.1016/j.biochi.2011.05.030 (2011). 21672603 10.1016/j.biochi.2011.05.030

[19] Gomez, B. & Štefanić, Z. Oligomeric symmetry of purine nucleoside phosphorylases. *Symmetry* **16**, 124. 10.3390/sym16010124 (2024).

[20] Kulikowska, E., Bzowska, Wierzchowski, J. & Shugar, D. Properties of two unusual, and fluorescent substrates of purine nucleoside phosphorylase: 7-methylguanosine and 7-methylinosine. *Biochim. Biophys. Acta* **874**, 355–363. https://doi.org/10.1016/0167-4838(86)90035-x (1986). 3098294 10.1016/0167-4838(86)90035-x

[21] Narczyk, M. et al. Single tryptophan Y160W mutant of homooligomeric *E. coli* purine nucleoside phosphorylase implies that dimers forming the hexamer are functionally not equivalent. *Sci. Rep.* **11**, 11144. 10.1038/s41598-021-90472-4 (2021). 34045551 10.1038/s41598-021-90472-4 PMC8160210

[22] Bzowska, A., Kulikowska, E. & Shugar, D. Properties of purine nucleoside phosphorylase (PNP) of mammalian and bacterial origin. *Z. Naturforsch.* **45c**, 59–70. 10.1515/znc-1990-1-211 (1990). 10.1515/znc-1990-1-211 2109978

[23] Bzowska, A., Kazimierczuk, Z. & Seela, F. 7-Deazapurine 2’-deoxyribofuranosides are noncelavable competitive inhibitors of *Escherichia coli* purine nucleoside phosphorylase (PNP). *Acta Bioch Polon*. **45**, 755–768 (1998). 9918502

[24] Kalckar, H. M. The enzymatic synthesis of purine ribosides. *J. Biol. Chem.* **167**, 477–486. https://doi.org/10.1016/S0021-9258(17)31000-1 (1947). 20285042

[25] Bzowska, A., Kulikowska, E., Shugar, D., Bing-Yi, C., Lindborg, B. & Johansson, N.G. Acyclonucleoside analogue inhibitors of mammalian purine nucleoside phosphorylase. *Biochem. Pharmacol.* **41**, 1791–1803. https://doi.org/10.1016/0006-2952(91)90117-n (1991). 1903945 10.1016/0006-2952(91)90117-n

[26] Bzowska, A., Kulikowska, E. & Shugar, D. Formycins A and B and some analogues: selective inhibitors of bacterial (*Escherichia coli*) purine nucleoside phosphorylase. *Biochem. Biophys. Acta* **1120**, 239–247. https://doi.org/10.1016/0167-4838(92)90243-7 (1992). 1576149 10.1016/0167-4838(92)90243-7

[27] Appleby, T. C., Mathews, I. I., Porcelli, M., Cacciapuoti, G. & Ealick, S. E. Three-dimensional structure of a hyperthermophilic 5’-deoxy-5’-methylthioadenosine phosphorylase from sulfolobus solfataricus. *J. Biol. Chem.* **276**, 39232–39242. 10.1074/jbc.M105694200 (2001). 11489901 10.1074/jbc.M105694200

[28] Zhao, H., Piszczek, G. & Schuck, P. SEDPHAT – A platform for global ITC analysis and global multi-method analysis of molecular interactions. *Methods Biocalorimetry* **76**, 137–148. 10.1016/j.ymeth.2014.11.012 (2015). 10.1016/j.ymeth.2014.11.012 PMC4380758 25477226

[29] Gasteiger, E. et al. Protein identification and analysis tools on the ExPASy Server BT. In *The Proteomics Protocols Handbook* (ed. Walker, J. M.) 571–607 (Humana Press, 2005).

[30] Krenitsky, T. A, & Tuttle, J. V. Correlation of substrate-stabilization patterns with proposed mechanisms for three nucleoside phosphorylases. *Biochim. Biophys. Acta* **703**, 247–249. https://doi.org/10.1016/0167-4838(82)90055-3 (1982). 6805517 10.1016/0167-4838(82)90055-3

[31] Bzowska, A. & Magnowska, L. Simple and universal method to determine dissociation constants for enzyme/ligand complexes. In *Nucleic Acids Symposium Series* Vol. 52 669–670 (2008). 10.1093/nass/nrn338 (2008). 10.1093/nass/nrn338 18776557

[32] Štefanić, Z., Narczyk, M., Mikleušević, G., Bzowska, A. & Luić, M. Crystallographic snapshots of ligand binding to hexameric purine nucleoside phosphorylase and kinetic studies give insight into the mechanism of catalysis. *Sci. Rep.*. 10.1038/s41598-018-33723-1 (2018). 30337572 10.1038/s41598-018-33723-1 PMC6193948

[33] Narczyk, M. et al. HpPNP shows new distribution patterns of open and closed active site conformations and unusual biochemical features. *FEBS J.*10.1111/febs.14403 (2018). 29430816 10.1111/febs.14403

[34] Bertoša, B. et al. Homooligomerization is needed for stability: A molecular modelling and solution study of *Escherichia coli* purine nucleoside phosphorylase. *FEBS J.* **281**, 1860–1871. 10.1111/febs.12746 (2014). 24785777 10.1111/febs.12746

[35] Narczyk, M. Biophysical basis of purine nucleoside phosphorylase from *E. coli* acting - mechanism of catalysis, relationship between protein function and its oligomeric structure. PhD Thesis, University of Warsaw (2019).

[36] Lee, J. et al. Expression, purification, and characterization of recombinant purine nucleoside phosphorylase from *Escherichia coli*. *Protein Expr Purif.* **22**, 180–188. 10.1006/prep.2001.1437 (2001). 11437593 10.1006/prep.2001.1437

[37] Jones, J. W. & Robins, R. K. Purine nucleosides. III. Methylation studies of certain naturally occurring purine nucleosides. *J. Am. Chem. Soc.* **85**, 193–201. 10.1021/ja00885a019 (1963).

[38] Ames, B. N. Assay of inorganic phosphate, total phosphate and phosphatases. *Methods Enzymol.* **8**, 115–118. https://doi.org/10.1016/0076-6879(66)08014-5 (1966).

[39] Brautigan, C. A. Fitting two- and three-site binding models to isothermal titration calorimetric data. *Methods* **76**, 124–136. 10.1016/j.ymeth.2014.11.018 (2015). 25484338 10.1016/j.ymeth.2014.11.018 PMC4591754

[40] Keller, S. et al. High-precision isothermal titration calorimetry with automated peak-shape analysis. *Anal. Biochem.* **84**, 5066–5073. 10.1021/ac3007522 (2012). 10.1021/ac3007522 PMC3389189 22530732

[41] Houtman, J. C. D. et al. Studying multi-site binary and ternary protein interactions by global analysis of isothermal titration calorimetry data in SEDPHAT: Application to adaptor protein complexes in cell signaling. *Prot. Sci.* **16**, 30–42. 10.1110/ps.062558507 (2007). 10.1110/ps.062558507 PMC1794685 17192587

[42] Kuzmic, P. Program DYNAFIT for the analysis of enzyme kinetic data: Application to HIV proteinase. *Anal. Biochem.* **237**, 260–273. 10.1006/abio.1996.0238 (1996). 8660575 10.1006/abio.1996.0238

[43] Wald, A. & Wolfowitz, J. On a test whether two samples are from the same population. *Ann. Math. Stat*. **11**, 147–162. 10.1214/aoms/1177731909 (1940).

[44] Burnham, K. P. & Anderson, D. R. *Model Selection and Multimodel Inference* (Springer, 2002).

[45] Richards, F. J. A Flexible growth function for empirical use. *J. Exp. Bot.* **10**, 290–300. 10.1093/jxb/10.2.290 (1959).

