# Supplementary material for: Multistep loss of catalytic and ligand binding abilities of hexameric purine nucleoside phosphorylase
Source: Sci Rep. 2026 Mar 2;16:11553. doi: 10.1038/s41598-026-41204-z (PMC13057136; doi:10.1038/s41598-026-41204-z)

# Supplementary Information

Multistep loss of hexameric purine nucleoside phosphorylase catalytic and ligand binding abilities

**Marta Narczyk*, Agnieszka Bzowska***

*Division of Biophysics, Institute of Experimental Physics, Faculty of Physics, University of Warsaw, Pasteura 5, 02-093 Warsaw, Poland*

## Table S1

| Exponential decay + line | | |
| --- | --- | --- |
| Parameter | Value | Standard Error |
| A_1_ | 147.96 | 24.51 |
| t_1_ | 56.7 | 5.6 |
| k | -0.024 | 0.003 |
| A_2_ | 73.28 | 1.43 |

Parameters fitted to the activity decay curve observed for the freshly thawed E. coli PNP stored at -25°C in 10 mM Tris buffer pH 7.4. Activity was determined at 25°C in 50 mM Tris-HCl, 50 mM Hepes-NaOH and 50 mM phosphate buffers, pH 7.6, adenosine was used a substrate. Function fitted to the data is defined in Materials and methods, equation (4).

Figure S1

SDS-PAGE of *E. coli* PNP samples with various specific activities.

Left panel: *E. coli* PNP sample just after purification on the affinity column, with the specific activity vs. inosine 103.2 ± 4.0 U/mg; left line, MW markers.

Right panel: *E. coli* PNP sample with the specific activity vs. inosine 75.0 ± 0.5 U/mg; line 1, MW markers; line 2, sample before the last purification step; lines 3-5, various concentrations of the purified *E. coli* PNP.


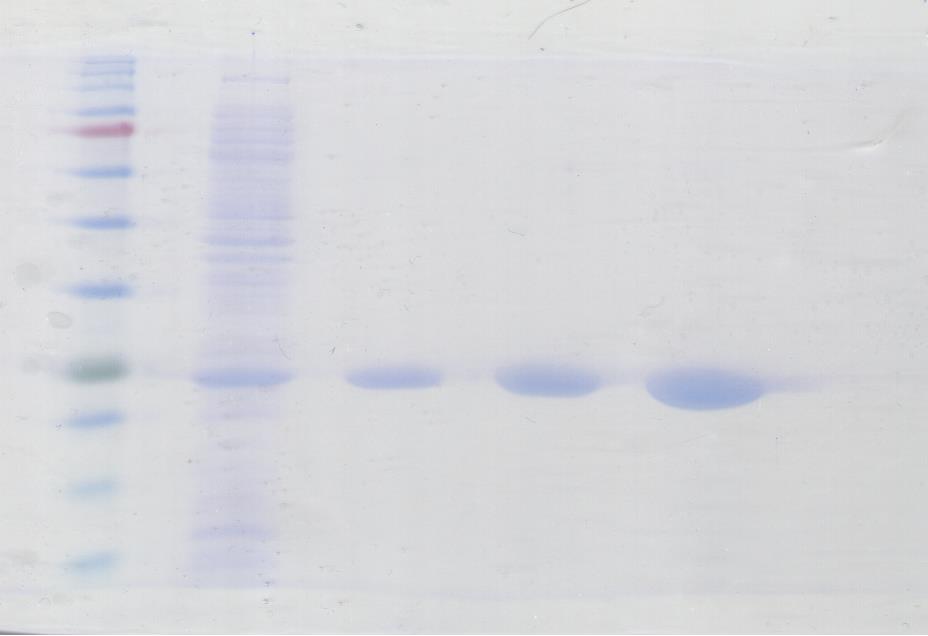


Marker 25 kDa →

Line 1 2 3 4 5


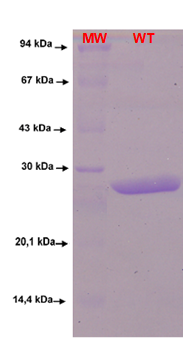


Table S2

## Specific activities of the *E. coli* PNP samples, for which a series of fluorimetric titrations with phosphate was done, and values of dissociation constants describing phosphate binding by the enzyme. Each series consists of five titrations, that were analysed globally. The three-binding-site model was fitted to the data because it best described the titration curves (see Methods).

| Specific activity *vs.* Ino  [U/mg] | Kd1  [μM] | Kd2  [μM] | Kd3  [μM] |
| --- | --- | --- | --- |
| 92.18 ± 2.20 | 0.60 ± 0.10 | 18.0 ± 1.6 | 10919 ± 2610 |
| 86.42 ± 1.34 | 1.68 ± 0.88 | 19.2 ± 7.8 | 8818 ± 2036 |
| 63.23 ± 1.86 | 2.15 ± 0.36 | 73.0 ± 6.3 | 1908 ± 96 |
| 59.80 ± 1.76 | 4.76 ± 0.40 | 125.6 ± 21.4 | 4468 ± 716 |
| 58.07 ± 1.56 | 13.40 ± 0.72 | 442.0 ± 25.3 | 15873 ± 2256 |
| 54.30 ± 2.35 | 3.39 ± 0.51 | 99.4 ± 9.9 | 2456 ± 166 |
| 54.03 ± 0.98 | 4.43 ± 1.11 | 84.4 ± 11.9 | 1774 ± 144 |
| 41.64 ± 3.41 | 15.25 ± 0.85 | 449.2 ± 36.6 | 10104 ± 1810 |
| 34.14 ± 1.85 | 6.02 ± 0.57 | 341.6 ± 27.7 | 53285 ± 9460 |
| 26.86 ± 1.34 | 6.63 ± 0.73 | 289.5 ± 28.2 | 16384 ± 1914 |
| 20.34 ± 2.25 | 9.74 ± 1.87 | 584.4 ± 112.1 | 11751 ± 2337 |
| 20.08 ± 2.57 | 15.25 ± 0.85 | 449.2 ± 36.6 | 10104 ± 1810 |
| 19.46 ± 0.10 | 28.92 ± 4.84 | 910.1 ± 180.7 | 90773 ± 21808 |
| 14.90 ± 1.91 | 5.94 ± 0.48 | 501.3 ± 43.5 | 45308 ± 6876 |
| 5.29 ± 0.31 | 964 ± 224 | 25203 ± 15846 | 55738 ± 18968 |

Table S3

Parameters obtained from fitting, with weights (see Materials and methods), a specific model to each of the activity decay curves (Figure 6) observed at 25°C for E. coli (part A) and H. pylori (Part B) PNPs in 50 mM Tris-HCl, 50 mM Hepes-NaOH and 50 mM phosphate buffers, pH 7.6. Fitted functions and meaning of each parameter are defined in Materials and methods, equations (2)-(6). The parameters characterizing the time of each phase observed in the activity decline profiles, are marked bold. The units of the parameters are the following: t [days], k [1/days], A [(U/mg)/days], h, p, q – no units (see eq. (2)-(6)).

| Table S3 A *E. coli* PNP | | | | | | | | | |
| --- | --- | --- | --- | --- | --- | --- | --- | --- | --- |
|  | Tris buffer pH 7.6 | | | Phosphate buffer pH 7.6 | | | Hepes buffer pH 7.6 | | |
| **Ino** | Line | | | Three-phase logistic function | | | Two-phase logistic function | | |
|  | Parameter | Value | Standard Error | Parameter | Value | Standard Error | Parameter | Value | Standard Error |
|  | A0 | 83.04 | 0.92 | A_∞_ | 11.19 | 5.19 | A_∞_ | -1.96 | 1.25 |
|  | k | **-0.028** | **0.005** | A_0_ | 68.48 | 61.67 | A_0_ | 134.34 | 28.31 |
|  |  | | | t_01_ | **19.0** | **1.6** | **t_01_** | **6.8** | **1.9** |
|  |  |  |  | t_02_ | **81.1** | **22.5** | **t_02_** | **61.5** | **1.0** |
|  |  |  |  | t_03_ | **227.2** | **121.2** | h_1_ | -0.015 | 0.003 |
|  |  |  |  | h_1_ | -0.92 | 45.44 | h_2_ | -0,070 | 0.011 |
|  |  |  |  | h_2_ | -0.012 | 0.012 | p | 0.73 | 0.05 |
|  |  |  |  | h_3_ | -0.009 | 0.011 |  | | |
|  |  |  |  | p | 0.11 | 0.22 |  |  |  |
|  |  |  |  | q | 0.46 | 1.47 |  |  |  |
| **m^7^Guo** | Line | | | Line | | | Two-phase logistic function * | | |
|  | Parameter | Value | Standard Error | Parameter | Value | Standard Error | Parameter | Value | Standard Error |
|  | A0 | 17.75 | 0.03 | A0 | 17.54 | 0.34 | A_∞_ | 10.96 | 8.36 |
|  | k | **-0.0014** | **0.0021** | k | **-0.002** | **0.004** | A_0_ | 24.33 | 22.62 |
|  |  | | |  | | | **t_01_** | **14.1** | **48.9** |
|  |  |  |  |  |  |  | h_1_ | -0.021 | 0.048 |
|  |  |  |  |  |  |  | A_∞_ | 0.07 | 0.59 |
|  |  |  |  |  |  |  | A_0_ | 15.54 | 0.72 |
|  |  |  |  |  |  |  | **t_02_** | **235.1** | **6.8** |
|  |  |  |  |  |  |  | h_2_ | -0.012 | 0.003 |
|  |  |  |  |  |  |  | Three-phase logistic | | |
|  |  |  |  |  |  |  | Parameter | Value | Standard Error |
|  |  |  |  |  |  |  | A_∞_ | -3.69 | 1.30 |
|  |  |  |  |  |  |  | A_0_ | 22.15 | 1.01 |
|  |  |  |  |  |  |  | t_01_ | 26.8 | 3.6 |
|  |  |  |  |  |  |  | t_02_ | 62.0 | 3.5 |
|  |  |  |  |  |  |  | t_03_ | 230.8 | 5.8 |
|  |  |  |  |  |  |  | h_1_ | -0.185 | 0.160 |
|  |  |  |  |  |  |  | h_2_ | 0.178 | 0.168** |
|  |  |  |  |  |  |  | h_3_ | -0.011 | 0.002 |
|  |  |  |  |  |  |  | p | 0.22 | 0.03 |
|  |  |  |  |  |  |  | q | 0.14 | 0.03 |

* preferred model, fitted separately for the range 0-56 days and 75-353 days.

**increase, i.e. positive h coefficient in a dose-response equation

| **Table S3 B *H. pylori* PNP** | | | | | | | | | |
| --- | --- | --- | --- | --- | --- | --- | --- | --- | --- |
|  | Tris buffer pH 7.6 | | | Phosphate buffer pH 7.6 | | | Hepes buffer pH 7.6 | | |
| *H. pylori* PNP | | | | | | | | | |
| **Ino** | Exponential decay + line | | | Two-phase logistic | | | Two-phase exponential decay | | |
|  | Parameter | Value | Standard Error | Parameter | Value | Standard Error | Parameter | Value | Standard Error |
|  | A_1_ | 19.79 | 1.30 | A_∞_ | 22.43 | 2.43 | A_∞_ | 1.02 | 0.20 |
|  | t_1_ | **25.3** | **4.7** | A_0_ | 38.18 | 0.73 | A_1_ | 62.86*** | 92.28 |
|  | k | **-0.056** | **0.011** | **t_01_** | **31.9** | **2.7** | t_1_ | **0.66***** | **0.66** |
|  | A_2_ | 16.36 | 1.57 | **t_02_** | **137.6** | **20.8** | A_2_ | 19.21 | 2.26 |
|  |  | | | h_1_ | -0.060 | 0.019 | t_2_ | **12.6** | **1.6** |
|  |  |  |  | h_2_ | -0.029 | 0.030 |  | | |
|  |  |  |  | p | 0.71 | 0.14 |  |  |  |
| **m^7^Guo** | Two-phase exponential decay | | | Exponential decay + line | | | Two-phase exponential decay | | |
|  | Parameter | Value | Standard Error | Parameter | Value | Standard Error | Parameter | Value | Standard Error |
|  | A_∞_ | 2.26 | 0.55 | A_1_ | 6.59 | 2.92 | A_∞_ | -0.2162 | 0.4698 |
|  | A_1_ | 13.14 | 5.69 | t_1_ | **3.30***** | **2.46** | A_1_ | 24.26*** | 13.17 |
|  | t_1_ | **1.92***** | **0.91** | k | **-0.008** | **0.004** | t_1_ | **1.02***** | **0.62** |
|  | A_2_ | 6.25 | 0.49 | A_2_ | 9.38 | 0.34 | A_2_ | 5.395 | 0.9453 |
|  | t_2_ | **53.5***** | **15.8** |  | | | t_2_ | **17.9** | **6.8** |

* big errors

Figure S2

Comparison of the secondary structure elements observed in the three-dimensional structure of *E coli* PNP and *H. pylori* PNP, red – α-helices, green – β-strands, blue – β-turns. Alignment was done for the structures of enzyme complexes with phosphate and formycin (for *E. coli* PNP PDB 4TS9 [32], for *H. pylori* PNP PDB 6F4X [33]).


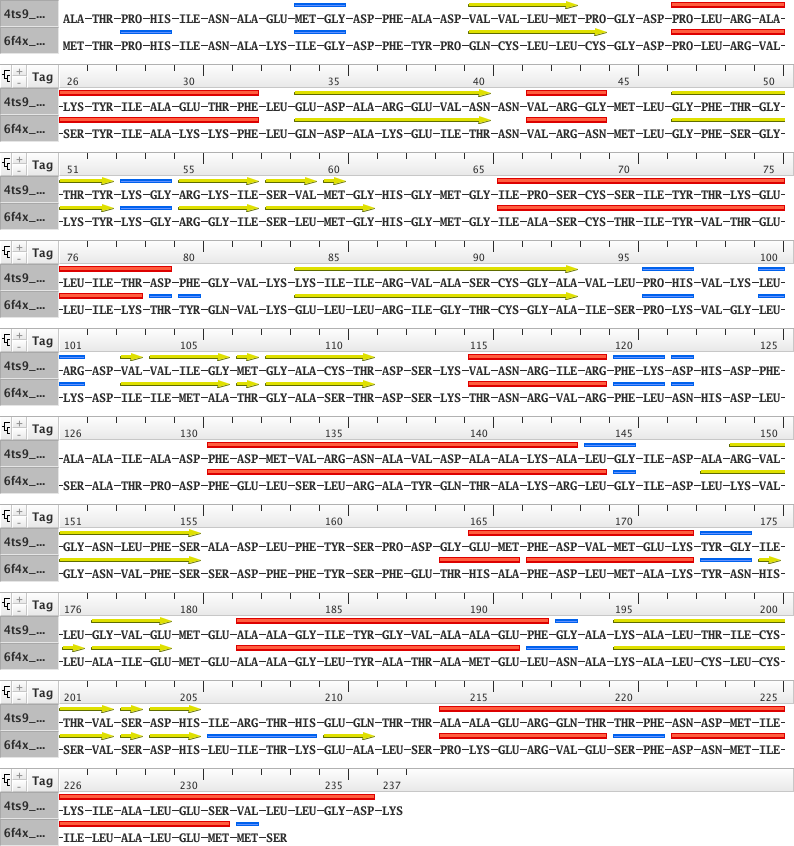

Supplement: Supplementary file 1 — Supplementary Material 1 [file 41598_2026_41204_MOESM1_ESM.docx]
